# Quantification of PEFC Catalyst Layer Saturation via In Silico, Ex Situ, and In Situ Small-Angle X-ray Scattering

**DOI:** 10.1021/acsami.3c00420

**Published:** 2023-05-25

**Authors:** Kinanti Aliyah, Christian Prehal, Justus S. Diercks, Nataša Diklić, Linfeng Xu, Seçil Ünsal, Christian Appel, Brian R. Pauw, Glen J. Smales, Manuel Guizar-Sicairos, Juan Herranz, Lorenz Gubler, Felix N. Büchi, Jens Eller

**Affiliations:** † Electrochemistry Laboratory, Paul Scherrer Institut, Villigen PSI 5232, Switzerland; ‡ Department of Information Technology and Electrical Engineering, ETH Zürich, Zürich 8092, Switzerland; § Photon Science Division, Paul Scherrer Institut, Villigen PSI 5232, Switzerland; ∥ Federal Institute for Materials Research and Testing (BAM), Berlin 12205, Germany

**Keywords:** polymer electrolyte fuel cell, water management, catalyst layer, representative morphology modeling, small-angle X-ray scattering

## Abstract

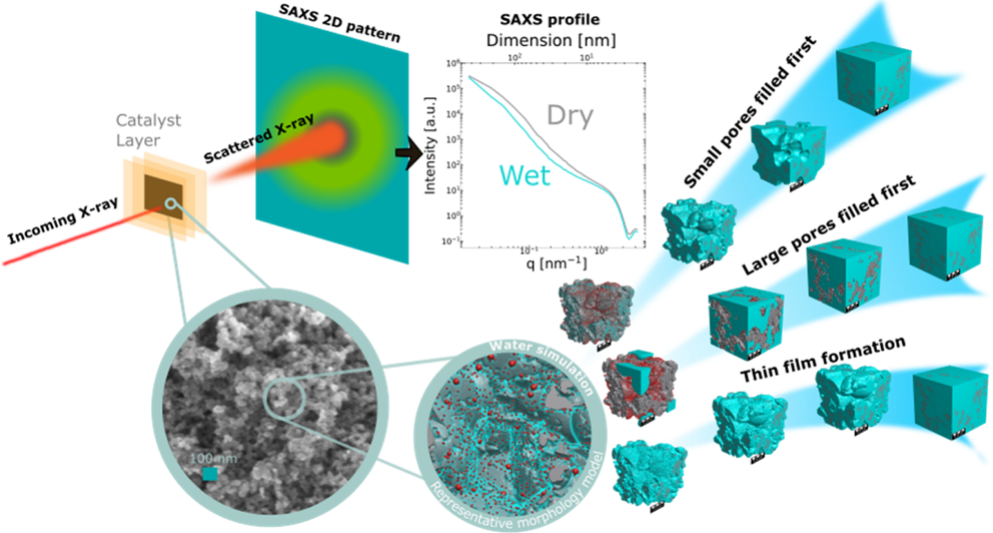

The complex nature of liquid water saturation of polymer electrolyte fuel cell (PEFC) catalyst layers (CLs) greatly affects the device performance. To investigate this problem, we present a method to quantify the presence of liquid water in a PEFC CL using small-angle X-ray scattering (SAXS). This method leverages the differences in electron densities between the solid catalyst matrix and the liquid water filled pores of the CL under both dry and wet conditions. This approach is validated using ex situ wetting experiments, which aid the study of the transient saturation of a CL in a flow cell configuration in situ. The azimuthally integrated scattering data are fitted using 3D morphology models of the CL under dry conditions. Different wetting scenarios are realized in silico, and the corresponding SAXS data are numerically simulated by a direct 3D Fourier transformation. The simulated SAXS profiles of the different wetting scenarios are used to interpret the measured SAXS data which allows the derivation of the most probable wetting mechanism within a flow cell electrode.

## Introduction

Water management is an important parameter for the operation of polymer electrolyte fuel cells (PEFCs) as the local oxygen transport resistance largely depends on the presence of liquid water.^1,2^ Excess of water can lead to liquid water accumulation in the gas diffusion layer (GDL), micro-porous layer (MPL), and catalyst layer (CL).^3^ Insufficient water content in the membrane electrode assembly (MEA) on the other hand can dry the polymer membrane, resulting in lower proton conductivity and creating higher ohmic losses.^4^

Over the last decade, a plethora of ex situ and operando characterization techniques have been utilized to study the accumulation of water in the porous structures of PEFCs (GDL, MPL, and CL).^5,6^ GDLs typically consist of carbon fibers forming pores in the size range from 18^7^ to 200 μm.^8^ Hence, these structures are well resolved by X-ray tomography using voxel sizes from 0.325–3 μm.^9,10^ Neutron imaging is also frequently employed because of its high sensitivity to water, but its spatial resolution of >5 μm sets constraints how the water distribution can be resolved.^11,12^

X-ray nano-tomography with a spatial resolution of 20 to 40 nm may contribute to directly resolve the water in the MPL and CL with pore sizes of up to 100 nm.^13^ However, it is difficult to employ this technique for operando measurements due to their limited field of view and because the ionomer in the MEA’s CL and membrane suffers significant radiation-induced chemical degradation.^14,15^

The materials in study usually are platinum/carbon-based porous structure with pore sizes less than 100 nm with some pores even smaller than the resolution of X-ray nano-tomography. They typically consist of porous carbon particles that act as the support for the catalytic Pt nanoparticles (customarily referred to as Pt/C) and a perfluorosulfonic acid ionomer which acts as a binder and proton conductor. The CL pores have a broad size distribution from primary inner-particle micro- and meso-pores (with diameters <2 vs 2–8 nm and wider, respectively) to inter-particle secondary pores (ranging from 10 to 200 nm). Low-surface area carbons (LSCs–such as Cabot Corp’s Vulcan XC72) have low internal porosity and typically support the majority of the Pt nanoparticles on the external carbon surface. In contrast, high-surface area carbon supports (like Akzo Nobel’s Ketjenblack EC600) possess high internal porosities and typically host the majority of the Pt nanoparticles inside their mesopores.^16^

Ex situ characterization of the Pt/C CL three-dimensional morphology has been carried out by various techniques. Focused ion beam scanning electron microscopy (FIB-SEM), both with^17^ and without^18^ preprocessing, has been explored down to 4 nm resolution.^19^ 3D transmission electron microscopy (TEM) has been employed to image carbon aggregates,^20^ Pt nanoparticles and the ionomer thickness on the carbon structure, using a contrast agent to distinguish the ionomer.^21^ Soft X-ray spectro-ptychography has also been used to image the ionomer distribution three-dimensionally.^22^

Despite the progress in describing the CL morphology, its relation with the water management in operative CLs has not been fully understood. Soboleva et al.^23^ have shown that for CLs based on different types of carbon support, ex situ water sorption and retention within the CL depend on their pore size distribution. A mathematical model for water adsorption and condensation in the CL has been developed and matched with experimental data by Mashio et al.^24^ To understand liquid and gas distribution in the through-plane direction of the CL, and how these affect the device performance, a simplified approach based on a two-phase pore-network model has been employed by Hannach et al.^25^ Simulating water saturation in the CL with a more realistic morphology, Sabharwal et al.^26^ used FIB-SEM reconstructed 3D volumes as a modeling reference to reconstruct CLs with varying porosities and pore size distributions without taking into account the Pt particles and the ionomer thickness. A lattice-Boltzmann method of wetting in artificial CL parameterized structures, completed with the ionomer phase, has also been investigated by Olbrich et al.^27^

To discern the water distribution across the various elements of an operating fuel cell, particularly the membrane and cathode/anode CL (s), water thickness across the MEAs can be extracted via transmission analysis from X-rays or neutron radiographs.^28^ Additionally, operando small-angle neutron scattering has been used to study water saturation in the CL albeit without time-resolved.^29^

Hence, to capture time-resolved insights, there is a need for characterization techniques able to reveal the transient liquid water saturation levels in the CL. Small-angle X-ray scattering (SAXS) is a good option as it is a powerful and fast technique to obtain bulk nano-morphological information. With SAXS, the morphology of the solid materials and their porous structure can be characterized on the nanoscale, for example, their porosity, tortuosity, pore size distribution, and structural changes.^30−32^ Thus far, SAXS has been used for studying mainly the change in the size of Pt nanoparticles in PEFC cathodes^33−35^ and to determine the morphology and hydration state of the membrane.^36−38^ Ultra-SAXS has also been employed to determine catalyst particle agglomeration in CL inks.^39^

Particularly challenging for SAXS data interpretation are porous structures that usually exhibit featureless SAXS curves, only of power-law decaying intensity with slight variation connected to its internal hierarchical structure.^40^ One of the analytical approaches is to describe the sample by an analytical solution of a simplified morphological model, such as polydisperse spheres or cylinders. More complex than elementary shapes, representative morphology structures with analytical solutions^31,41,42^ like a two-phase alloyed metal have also been employed. Another alternative is to simulate the scattering of an object described by its outer envelope using point cloud scattering.^43,44^ For quantifying the change in electron density of the component, a model-independent invariant calculation is typically used.^45^

**Figure 1:**
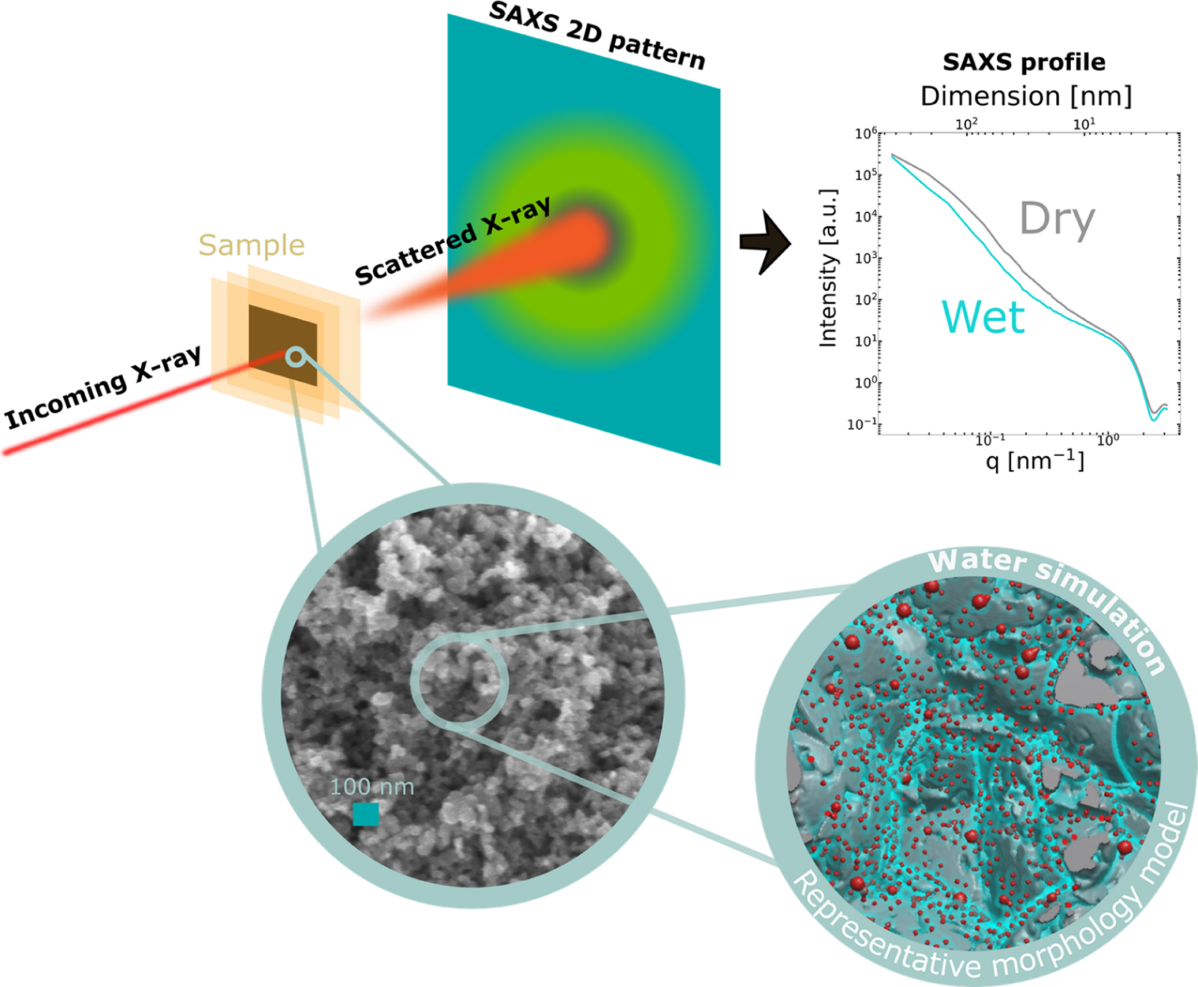
SAXS relies on X-ray photons interacting with the electron density heterogeneities of a sample, the photons then scatter (top left) and arrive onto the collection on a 2D detector plane (top middle). Following corrections and azimuthal averaging, a 1D intensity profile versus scattering vector q can be obtained (top right). By means of a representative hydrated morphology model (bottom right) mimicking the real structure of the porous CL (SEM of Pt/C, bottom left), the effect of the presence of water on the structure and the in silico scattering behavior can be investigated (top right).

Multi-phase structures (meaning that a material with a number of phases >2) scattering length densities within an order of magnitude can be difficult to interpret. This is due to the lack of analytical solution presence, or the emergence of cross-correlations terms complicating the information derivation. Here, the scattering from the 3D electron density map of the object can be used to aid the interpretation. As the SAXS intensity is proportional to the square of the electron density contrast and to the volume fraction of the sample components, a change in the composition or its liquid saturation will be registered by the SAXS data, as schematically described in Figure 1. This effect has already been leveraged and interpreted by representative structure modeling in other energy-related fields such as battery electrodes.^46^

Here, we show, as a proof-of-principle, the use of SAXS to determine the water saturation level in multiphasic PEFC CLs. For data interpretation, we generate a representative 3D morphology by fitting of stochastic structure models to SAXS data. In silico scattering data of the representative 3D structures at varying saturation levels and assuming different water filling mechanisms are calculated numerically. We show that the effect of the water on the structure in the CL pores and on the SAXS intensity can be captured by this numerical approach. The detectability of liquids inside the porous materials by SAXS is also verified by ex situ wetting experiments of a model material, a non-noble metal catalyst (NNMC), and Pt/C CL. To quantify the water saturation, a model-free invariant calculation is used complementarily to the in silico scattering approach. The methodology is finally applied to investigate in situ the wetting of a Pt/C CL in a liquid electrolyte flow cell.

## Experimental Section

### Materials

#### NNMC Catalyst Layer

The NNMC used in this work was prepared following the procedure in reference^47^ (referred as wet BM-3 mm in the publication). Briefly, polyacrylonitrile (PAN, Sigma-Aldrich) and sodium carbonate (Na_2_CO_3_, Sigma-Aldrich, anhydrous, 99.999% trace metal basis) were used as the C- and N-precursor and pore inducing agent, respectively, while Fe^2+^ acetate (Sigma-Aldrich, >99.99% trace metal basis) pre-complexed with phenanthroline (Sigma-Aldrich, >99%) was used as the Fe-precursor. First, PAN and Na_2_CO_3_ were stirred separately (in a mass ratio of 1:2) overnight in dimethylformamide (DMF, Sigma-Aldrich, anhydrous 99.8%—in a Na_2_CO_3_/PAN/DMF weight ratio of 1:95) at 80 °C. After mixing and stirring these two solutions for another 1 h at 80 °C, Fe^2+^:phenantroline complex prepared in DMF (with a molar ratio of 1:5) was added into the PAN and Na_2_CO_3_ mixture, in which the Fe content in the initial mixture corresponded to 1 wt % on the basis of all precursors’ masses. Afterward, the solvent was evaporated overnight in a furnace at 80 °C for complete dryness. 1.5 g of the resulting dry powder underwent wet-milling (planetary ball mill, Fritsch, Pulverisette, 45 mL) with 3 mm diameter ZrO_2_ balls with a ball-to-precursor-mixture mass ratio of 27 by adding 10 mL of ethanol (VWR, ≥99.8%, AnalaR NORMAPUR) for 16 h. Afterward, the resulting slurry was passed through a 100 μm sieve and dried. The resulting ground powder was then submitted to a first heat treatment (HT) in N_2_ (Messer AG, 5.0–200 mL·min^–1^) at 700 °C for 1 h, that was followed by acid treatment in 1 M sulfuric acid (H_2_SO_4_, Merck, 95–97%) at 80 °C for 4 h. Lastly, the acid-washed powder was submitted to a second HT step in 5% H_2_ [balanced with Ar, Pangas (5.0)–200 mL·min^–1^] at 950 °C for 40 min.

To fabricate the NNMC CL, a catalyst ink was prepared by dispersing the NNMC in a mixture of iso-propanol and ultrapure water with the volume ratio of 3:7, as to yield a suspension with a solid-to-liquid content of 9 mg_NNMC_/mL_IPA+H2O_. Then, Nafion solution (5 wt % EW: 1100, Sigma-Aldrich) was added to the dispersion to yield a Nafion-to-catalyst mass ratio of 0.2. After the resulting ink was sonicated for 30 min, the NNMC layer was fabricated by hand-spraying on 25 μm thickness Kapton Type 100 HN Film.

#### Pt/C Catalyst Layer

The Pt/C catalysts for ex vs in situ experiments with 50 wt. % Pt on Vulcan XC-72 carbon (Pt/V 50% wt Pt) or a 30 wt % Pt on Vulcan XC-72 carbon (Pt/V 30% wt Pt) were both purchased from Tanaka Kikinzoku Kogyo Co. (product codes TEC10V50E and TEC10V30E, respectively).

Weighed amounts of each powder were diluted in a mixture of ultrapure water (18.2 MΩ cm, prepared by ELGA Purelab Ultra), isopropanol (IPA, 99.9% Chromasolv Plus for HPLC, Sigma-Aldrich), and ionomer (≈5 wt % Nafion EW/1100, Sigma-Aldrich) in 6:5.1:2.4 or 3:1:0.4 volume ratios (for ex situ vs in situ measurements, respectively) and ultrasonicated to produce inks that were spray-coated on 25 μm thick Kapton Type 100 HN Film (ex situ) or on Kapton 200RS100 (in situ).

For the spray coating (Sonotek, ExactaCoat, with an ultrasonic nozzle), the inks were loaded into a syringe containing a magnetic stirrer. The Kapton foils were fixed in polyoxymethylene frames and placed on a 60 °C hot plate. The inks were sprayed onto the Kapton films with an ink flow rate of 0.05 mL/min, a nozzle height of 10 mm, and a nozzle speed of 80 mm/s.

Information from the ink formulation and thickness for SAXS fitting parameters is presented in Table 1. The thickness was measured with a portable profilometer in five different spots and averaged. The porosity ε was calculated by
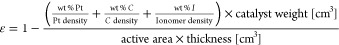
1

**Table 1:** Parameters of the Ink Formulations and the Resulting Catalyst Layers

	ex situ NNMC	ex situ Pt/C	in situ Pt/C
thickness [μm]	32	5	77
active area [cm^2^]	1	1	0.145
ionomer-to-carbon mass ratio (ICR) [−]	0.2	0.6	0.33
catalyst-loading [mg_Pt_/cm^2^]/[mg_NNMC_/cm^2^]	0.8	0.26	1.43
Pt vol. fraction [%]	0	2.44	0.85
carbon vol. fraction [%]	11	28	20
ionomer vol. fraction [%]	3	18	7
total solid fraction [%]	14	48	28
porosity [%]	86	52	72

Volume fraction and porosity values are based on materials’ particle density of 21.65, 2.1, and 1.98 g/cm^3^ for platinum, carbon, and Nafion ionomer, respectively. The weight percentage in eq 1 is the weight percentages of the individual components normalized by the total weight of all components in the CL. The high-loading CL (∼1.5 mg_Pt_/cm^2^) was used in the in situ experiment to achieve good through-plane signal from the sample as proposed earlier.^33,35^ A realistic fuel cell application usually requires a lower loading (∼0.1–0.4 mg_Pt_/cm^2^). A low ionomer-to-carbon mass ratio (ICR) was notably employed for NNMC to reduce effects of ionomer degradation due to radiation. For Pt/Vulcan inks, the optimized ICR is 0.6, hence the ICR of ex situ Pt/C wetting.^48^ For the in situ wetting, probing many times the same CL spot by X-ray, we also used lower ICR to minimize artifacts coming from ionomer swelling and from potential degradation of the ionomer.

#### Hydrophilic Porous Membrane

Durapore hydrophilic PVDF biological membrane filters VVLP04700 (Merck Millipore Ltd.) were used as model materials because their pore size (≈0.1 μm) and porosity (≈70%) values are similar to those of a typical PEFC CLs.

#### Wetting Liquids

For ex situ wetting experiments, *n*-decane bought from Thermo Fisher/Fisher Scientific AG (purity ≥99%, Switzerland) was used for achieving complete saturation of the Pt/C CL. Ultrapure water (18.2 MΩ·cm, ELGA Purelab Ultra) was used whenever water is mentioned.

For in situ wetting experiments, 0.1 M HClO_4_ electrolyte was used and prepared from 70% HClO_4_ (VERITAS DOUBLE DISTILLED, GFS Chemicals) diluted in ultrapure water (18.2 MΩ·cm ELGA Purelab Ultra) and saturated with N_2_ (Carbagas AG, purity 5.0 from beamline gas line) during the whole measurement time.

## Methods

### Experimental Setup

#### Ex Situ Wetting

CLs spray-coated on 25 μm thick Kapton foil substrates as well as hydrophilic porous membranes were placed into Kapton pockets with 25 μm wall thickness to avoid liquid evaporation. The samples were mounted perpendicular to the beam direction (see Figure 1 left for scheme and Supporting Information Figure S1a). Samples were measured in dry state, as well as wetted with water (PVDF membranes) or *n*-decane (CLs).

#### In Situ Wetting

In situ wetting was realized in a spectroelectrochemical flow cell originally designed by Binninger et al.,^35^ previously employed in the in situ characterization fuel cell and electrolyzer catalysts (see a schematic representation of the experimental setup in Figure S1b).^33,49,50^ The cell was mounted as to orient the working electrode substrate (Pt/C) in perpendicular orientation into the beam. A carbon black-based counter electrode is located not in the beam path to avoid any signal bias stemming from the counter electrode. The nitrogen saturated electrolyte was flowing from bottom of the cell to outlet on the upper part of the cell with a flow rate of 50 μL/min to enable potential-induced pseudo-capacitive reactions (Pt–oxidation, Pt–O reduction, H adsorption, and H desorption in the working electrode). Electrochemical protocols were carried out with EC-Lab (BioLogic) software and a Biologic potentiostat SP-300. The protocol consisted of the following steps: (1) measuring the open-circuit voltage (OCV) until its value stabilized; (2) recording a linear potential sweep from the OCV to 0.4 V_RHE_; (3) recording four cyclic voltammograms (CVs) at 50 mV/s from 0.05 to 1.1 V vs RHE, six CVs at 50 mV/s from 0.05 to 1.2 V vs RHE, and three CVs at 10 mV/s from 0.05 to 1.1 V vs RHE; (4) 5 min potential holds at 0.4, 0.1, 0, and −0.1 V vs the reversible hydrogen electrode (RHE) and, in between the potential holds, recording of three CVs at 10 mV/s from 0.05 to 1.1 V vs RHE. The Pt utilization was calculated by deriving the hydrogen underpotential deposition (*H*_upd_) charge from the CVs recorded in between potential holds (Figure S9) and normalizing that charge with the value extracted from a separate measurement of *H*_upd_ performed on a thin film of the same Pt/C catalyst (with a loading of 10 μg_Pt_·cm^**–2**^) on a glassy carbon disk in a rotating ring disk electrode setup in the same electrolyte.

### Scanning SAXS Measurement Pattern and Data Reduction

#### Lab-Source Ex Situ Catalyst Layer

The SAXS/WAXS measurements were conducted using the Methodology Optimization for Ultrafine Structure Exploration (MOUSE) protocols.^51^ An accessible q-range is 0.01408 < *q* < 24.15 nm^–1^. X-rays were generated from microfocus X-ray tubes, followed by multilayer optics to parallelize and monochromatize the X-ray beams to wavelengths of Cu Kα (λ = 0.154). Scattered radiation was detected on an in-vacuum Eiger 1M detector (Dectris, Switzerland), which was placed at multiple distances between 138 and 2507 mm from the sample. The resulting data have been processed using the DAWN software package in a standardized complete 2D correction pipeline with uncertainty propagation.^52,53^

#### Synchrotron-Source Ex Situ Catalyst Layer Wetting

A horizontal line scan consisting of 100 measurement spots and with a step size of 0.1 mm was recorded at the cSAXS beamline of the Swiss Light Source, Paul Scherrer Institut, Switzerland. The beam size was 0.1 *×* 0.1 mm^2^, and 0.05 s exposure time was used for each spot.

#### Synchrotron-Source In Situ Catalyst Layer Wetting

SAXS measurements were conducted at the cSAXS beamline. The spot size on the sample as measured by scanning a sharp edge was 7 μm vertically and 28 μm horizontally.

To exclude spatial heterogeneity in the CL, four points separated by 100 μm in horizontal, 50 μm in vertical were measured to probe a representative area of 784 μm^2^ of the CL. In total, four SAXS profiles were recorded for each condition with an exposure time of 0.1 s. Due to overhead time in between motor movements, the first of four points to the first of next four points are 6 s apart.

#### For Both Ex Situ and In Situ Synchrotron Source Wetting

An evacuated flight tube was placed between sample and detector to reduce air scattering. SAXS profiles were recorded with a Pilatus2M detector^54^ with a sample-detector-distance of 7.128 m. An X-ray energy of 11.2 keV was selected using a Si(111) double crystal monochromator, and the beam was focused on the sample by the combination of a horizontally focusing monochromator crystal and a vertically focusing mirror. An accessible *q*-range is 0.01359 < *q* < 1.676 nm^–1^. Data reduction was performed with cSAXS processing scripts.^55,56^ Recorded azimuthally integrated 1D intensities, containing scattering from samples and kapton container, were normalized by the transmission, measured with an Oxford Danfysik CyberStar. Then, we subtracted the normalized background signal measured from an empty container to obtain the scattering of the sample. Intensities were calibrated to absolute units where specified, and the absolute beam intensity was calibrated using a standardized glassy carbon and normalizing by the thickness of the sample. A constant background of the intensity (*C*) was subtracted by first fitting 10 points at high *q* (*q* > 1 nm^–1^) to *Aq̂* – 4 + *C*, where *A* is slope of the power law.

### SAXS Data Interpretation Tools

#### *Determination* of the Liquid Saturation Level by Invariant Calculation

Calculating the invariant of the scattering intensity *I(q)* allows quantifying the amount of substances in the irradiated volume of the sample. The integrated intensity or invariant *Ĩ* of the experimental SAXS intensity (ideally in absolute units) is numerically calculated by
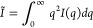
2

This implies an extrapolation to *q* = 0 and *q* = ∞ and a sufficiently broad data *q*-range. To perform this computation, at low *q*, the SAXS intensities may be extrapolated using the Guinier model *I(q)* = *I*(*0*)*ê*(*−q*^2^*Rg*^2^/3*)*, where *Rg* is the radius gyration and *I*(*0*) is the intensity approximated at *q* = 0; and at high-*q*, with the Porod law*, I*(*q*) = *Aq̂* – 4*.* However, in our experiment, we do not observe a Guinier-like scattering regime at low *q*. Therefore, we calculated the relative change of the invariant from the experimentally limited *q*-range, which still allows us to calculate an approximate value for the liquid saturation level within pores of relevant sizes (1–100 nm). The accuracy of this approach depends on the shape of the measured SAXS intensities, as discussed in the Results and Discussion section.
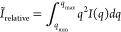
3

For the materials of interest shown in this paper, a multiphase description of the invariant is needed. For the partially saturated hydrophilic porous membrane case, the three phases are the solid porous membrane, void, and liquid. In case of the partially saturated Pt/C CL, five different phases exist: carbon, Pt, ionomer, void, and liquid. SAXS interpretation involving five phases will be complicated due to the cross-term relations; hence, a simplification where applicable must be carried out.

**Figure 2:**
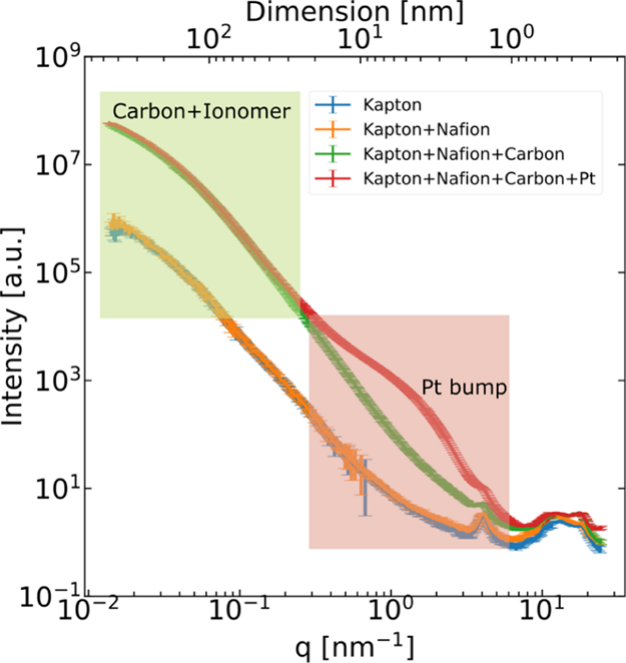
SAXS measurements of Kapton substrate, Kapton + Nafion ionomer layer, Kapton + Nafion + carbon, and Kapton + Nafion + carbon + Pt with the same CL fabrication method and ink as in situ wetting material, deconvoluting the contribution of the components in the Pt/C CL; top axis is real space dimension D corresponding to 2π/*q*.

Since the contribution of a pristine ionomer layer sprayed on Kapton was insignificant compared to combined carbon and ionomer (will also be explained in the Results section, see Figure 2), the invariant calculation of the different phases could be simplified. The five-phase system could thus be reduced to a four-phase system by assuming that the ionomer is part of the carbon phase. Given nearly identical electron densities between ionomer and carbon, such an volume-averaging approach was feasible.^57^ This simplification was also applied to the invariant calculation of the scattering of simulated structures and for the non-noble metal CL. Note that when ionomer is wetted by water, the electron density will change, and the thin ionomer layer will swell.^58^ This approximation holds true when CL is dry or wetted by *n*-decane. The assumption was used throughout this work. In the future, the ambiguity of ionomer in CL is wetted by water causing a decrease in electron density, versus water filling the pores may be investigated and taken into account as water saturation level margin of error.

The four-phase system of Pt/C was further reduced to a three-phase system by assuming that the Pt contribution was a constant. There are two strategies on how to subtract the contribution of Pt–assuming analytical solution of polydispersed spheres, or Guinier-Porod fit to Pt bump (0.6 < *q* < 2 nm^–1^). For experimental data, *q* range of Pt is fitted with an analytical expression for scattering of polydisperse spheres to a log–normal distribution, as typically carried out in the literature.^33^ For SAXS data from the simulated structure, the constant contribution was carried out by fitting with Guinier law at the Pt bump as the 3D array to create Pt/C structure is pixelated and not enough to resolve angstrom precision (see Figure S2). The simplification of Pt contribution to a constant as opposed to having correlation with other phase is feasible because of Pt’s significantly larger electron density. Cross-correlation term of Pt and C is assumed to be negligible, which might not be the case as revealed in anomalous SAXS.^59^ The assumption leads three-phase approximation of which the invariant calculation is easier to handle. The invariant for three-phase was used for both the wetted Pt/C and wetted NNMC CLs. It is important to note that invariant calculation is model-free and solely for quantification. It could be carried out without simulated structure and/or analytical solution fit to experimental data, hence is easier to implement. However, invariant calculation could not be used to derive insights into the *q*-dependent pore-filling mechanism.

The equations for the multiphase invariant calculation can be found in the Supporting Information. A similar approach to the aforementioned invariant calculation has been applied to both the experimental as well as the simulated data.

### SAXS Model Fits for Pt/C and NNMCs

In order to obtain morphological parameters, to feed into the 3D structure generations from the 1D SAXS intensity profiles, the experimental SAXS data were fitted using an intersected Boolean model (IBM).^32,42,46^ The fitting is carried out within the *q*-range where the carbon support scattering contribution dominates, most prominent from 0.01 < *q* < 0.1 nm^–1^ for Pt/C and 0.01 < *q* < 1 nm^–1^ for the NNMC. The IBM^42,46^ can account for the fractal-like nature of the Pt/C and NNMC CLs. One class of the Boolean model is defined as randomly distributed spherical grains with a specific radius and defined volume fraction. The IBM uses multiple classes of Boolean models whose final form is from the constructive solid geometry intersection of the total.

To fit the data of the Pt/C CL, eight classes of IBMs were used, resulting in a total of 17 free parameters (eight radii, eight volume fractions, and one relative scaling factor). For the NNMC CL, 10 classes of IBMs were used, thereby a total of 21 free parameters (10 radii, 10 volume fractions, and a scaling factor). In order to optimize the parameters, we used the “Evolution Strategy” optimizer from the Hyperactive package^60^ with the following cost function
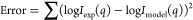
4

A penalty on the error is applied when the radius of class *i* is smaller than the radius of class *i*–1*,* imposing that the subsequent class’ radius being larger than the previous class.

The calculation details for the analytical fitting using the IBMs are given in the Supporting Information. The code (gitlab.psi.ch/fcsaxs/fcsaxs) is extendable to as many Boolean classes as desired; the more classes of IBM are parameterized, the more control of structural hierarchy could be obtained. Fitting takes about 5 h for 1000 iterations on an Intel(R) Xeon(R) CPU E5-2687W v3 @ 3.10 GHz with 10 cores.

#### Stochastic Generation of Carbon + Ionomer 3D Structures for Both Pt/C and NNMC Catalyst Layers

The fitting parameters from the analytical IBM solution are subsequently used to generate a statistically representative structure of the carbon + ionomer solid in 3D. The structure generation code is built on a function in the Porespy package^61^ for generating overlapping spheres which constitute the different Boolean classes. First, we simulate *i* 3D arrays (representing *i* classes) and populate them with overlapping spheres of a given radius and volume fraction obtained from the fit of each class. Each voxel that is assigned to a sphere has a value of 1, the remainder take the value of 0. Next, the values of all voxels in all simulation boxes were summed up in a new simulation box. Only voxels with values equal to total number of classes, *i*, were kept as solid, whereas voxels with a value less than *i* were assigned to a value for pores in the new simulation box. Due to the stochastic nature of the model, the iterative process is needed when the desired porosity is not reached. For Pt/C, 512^3^ is used as simulation box size, defined as the size of a 3D array used, and for NNMC, 1024^3^ is used (bigger dimension is used to accommodate a wider fractal nature across *q*-range compared to Pt/C from the power law exponent in SAXS data, see the Results section).

#### Addition of Pt on the Carbon + Ionomer 3D Structures with a Constraint of Matching Both Dry and Fully Wet Structure (Only for Pt/C)

After the creation of the carbon + ionomer 3D structure in the previous step, Pt nanoparticles need to be added to the structure to simulate the Pt/C CL. Careful consideration of the amount and placement of the Pt to properly match both the dry and fully wetted Pt/C is needed. Therefore, a “loading region” is defined, in which placing a Pt nanoparticle is allowed. This region is created by 2*r* + 1 binary dilation of the original carbon + ionomer structure, where *r* is the radius of the Pt nanoparticles intended to be. In this way, the Pt nanoparticles are surely placed on the surface of the carbon + ionomer structure. In reality, it is not a realistic way to have Pt on top of the ionomer + carbon structure. The Pt should be placed below the ionomer as the ionomer is only incorporated when making CL out of Pt/C catalyst powders. A way to represent this discrepancy from reality could be by burying Pt few nanometers (an additional input parameter) from the carbon + ionomer surface; however, it is not implemented in this work for simplicity.

A “pore size threshold” can also be introduced such that if used, pores with sizes below the threshold do not have Pt (as illustrated in Figure S4). This makes the Pt particles more concentrated in certain structures and introduces an additional scattering contribution due to the long-range Pt–Pt particle interaction. The “loading region” depending on the Pt radius, the “pore size threshold”, and the Pt radii (2, 3, 4 nm, only steps of 1 nm is possible since the simulation box voxel size is 1 nm) with the corresponding volume fractions are defined as fitting parameters. An optimization procedure taking into account both wet and dry data minimizes the error in eq 5 to obtain the parameters well matching the experimental data of simultaneously fully wet and dry Pt/C. *I*_exp,dry/wet_(*q*) is SAXS intensity of experimental dry/wet, and *I*_img2sas,dry/wet_(*q*) is SAXS intensity of simulated structure dry/wet.
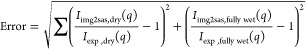
5

For the two-phase CL (without Pt) or NNMC, this procedure is not necessary as there is already an excellent match between experimental data and the simulation (both in dry and fully wet conditions) using the analytical Boolean fit and the subsequent 3D image generation. Fitting takes about 16 h with 200 iterations on an Intel(R) Xeon(R) CPU E5-2687W v3 @ 3.10 GHz with 10 cores.

##### SAXS Profiles Obtained from the 3D Structures (IMG2SAS)

To obtain the scattering intensities from 3D structural inputs, the code IMG2SAS was developed in Python, adapted from previous efforts in the literature.^62,63^ The code can accept any input in the form of 3D Numpy arrays, which can be synthetic images from, for example, imaging-based Python packages such as Porespy, 3D images measured by either FIB-SEM, transmission electron microscopy (3D TEM), or ptychographic X-ray nanotomography. Within this work, artificial structures are used to analyze SAXS data.

The algorithm consists of applying a 3D fast Fourier transform (FFT) to the 3D electron density map of a sample, shifting the zero frequency of the 3D FFT to the center, taking a square of real components of the FFT added to square of the imaginary components, which together constitute 3D intensity, and finally spherically averaging the intensity. The time to run this code largely depends on the 3D image input size. IMG2SAS has been verified for basic shapes, such as spheres, core–shell particles, cylinders, cubes, and porous structures with spherical pores by comparing the SAXS profiles from IMG2SAS to the expected analytical solution. The code for the IMG2SAS is in gitlab.psi.ch/fcsaxs/fcsaxs, further details and test studies are available in the Supporting Information.

### Water Simulation on the Representative Morphological Model

Here, three filling modes inspired from morphological operations are introduced and used to describe the formation of liquid water in an operating FC and during the filling of the CL pores of the in situ wetting experiment, namely (a) small-pores–wetted-first, (b) large-pores-wetted-first, and (c) thin-film formation. We first create a 3D map of local pore sizes. The Porespy^61^ function of “local thickness” is used to calculate the radius of the largest sphere that (1) engulfs the voxel and (2) fits entirely within the pore space for each voxel. The voxel is then labeled by the size of the aforementioned largest sphere. The function outputs a 3D array with values containing the biggest pore size fit of each pore voxel. The pore size map from local thickness calculation is needed for the former two filling modes.

For the small-pores-first scenario, a pore size threshold is applied, where pore voxels with values below or equal to the threshold of the local pore size map are assigned to liquid water. After weighing all carbon/ionomer voxels with the corresponding scattering length density (SLDs), the scattering intensities can be calculated using IMG2SAS. An invariant calculation determines the saturation level and verifies the saturation level as determined by counting water-filled voxels. The large-pores-first algorithm works equivalently; the pore size threshold starts from the biggest pore size and gradually decreases where voxels above pore or equal to the threshold are assigned to water. To mimic water thin-film formation, a binary dilation is applied to the original 3D dry structure with increasing iterations to represent increasing thin-film water thickness. The code to determine the local pore size map, the simulation of the different wetting scenarios, and the evaluation of the saturation levels from voxel counting and invariant are available in gitlab.psi.ch/fcsaxs/fcsaxs.

## Results and Discussion

In this section, a systematic approach toward PEFC CL saturation determination is presented. First, the different contributions of Pt/C CL components making up the whole Pt/C CL were elucidated by examining their corresponding SAXS profiles. Then, the detection of liquid in porous structures (models of CL with similar pore size, non-nobel metal CL, and Pt/C CL) was investigated. Establishing different well-known saturations experimentally is difficult. Therefore, we put our best effort in the decane/water wetting of the porous layers to show sensitivity for dry and 100% saturation. We chose porous PVDF and NNMC catalysts as model systems that have similar pore sizes and aiming more and more to the complexity of Pt/C CLs. A representative morphology model simulating different wetting scenarios between dry and 100% saturation in a Pt/C CL was developed to aid the data interpretation. Finally, in situ filling of Pt/C was interpreted by derived methods, i.e., the invariant calculation and representative morphology model.

### Ex Situ SAXS of Different Components of the Pt/C Catalyst Layer

To understand in which *q*-range and to which significance each component in the Pt/C CL is mainly contributing, four different sample compositions were measured–Kapton substrate as background, Kapton + ionomer, Kapton + ionomer + carbon, and Kapton + ionomer + carbon + Pt nanoparticles (see Figure 2). The ink recipe of the whole components measured was the same as the CL used in the in situ experiments.

The contribution from only the ionomer thin film in orange was much smaller (only in WAXS region of *q* > 1 nm^–1^) than the broad contribution of the Pt-free carbon electrode (carbon and ionomer). The SAXS intensity profile (intensity in arbitrary unit versus scattering vector *q*, in a log-log scale) of the Pt/C-electrode (Kapton + carbon + ionomer + Pt) deviated further by forming a “Pt-bump” at *q* ≈ 0.15 to 1 nm^–1^ due to the Pt nanoparticles.

### Ex Situ Wetting of Model Material by Liquid Water

**Figure 3:**
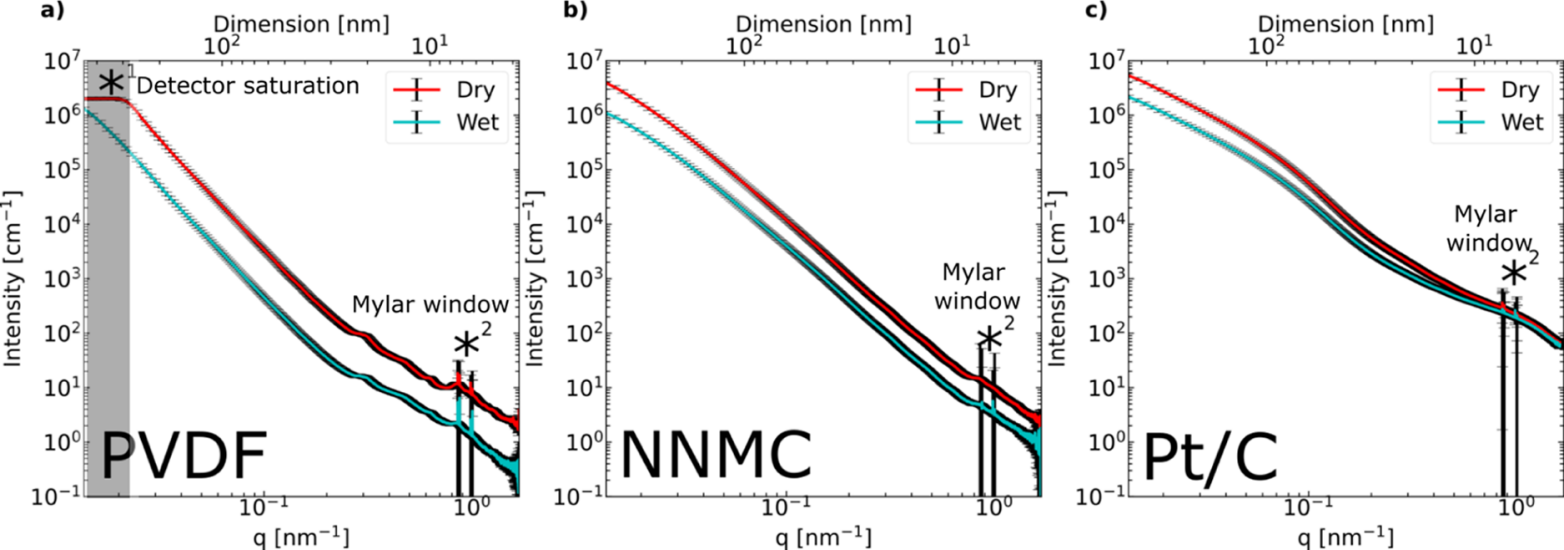
Azimuthally averaged SAXS intensity versus scattering vector q of (a) porous PVDF membrane dry and wetted by water, (b) non-noble metal CL dry and wetted by *n*-decane, and (c) Pt/C CL dry and wetted by *n*-decane; top axis is real space dimension D corresponding to 2π/q.

At first, the detectability of liquid water by SAXS was evaluated using a model material. A porous hydrophilic PVDF membrane with a porosity of ≈70% and a pore size of ≈100 nm within the order of magnitude of the pores in PEFC CLs was chosen to be fully wetted with liquid water. The SAXS 1D profiles of this model material in dry and wetted states are shown in Figure 3a. The SAXS profiles show *I* ∼ *q*^–4^ for both dry and wet materials in the *q* region 0.02 < *q* < 0.2 nm^–1^, indicating a smooth interface^64^ for the real-space dimension of ≈31 < *D* < 314 nm. In addition, the peaks visible in the *q* region 0.2 < *q* < 1 nm^–1^ may be attributed to the nanostructure of crystalline domains of PVDF which is expected to be in *q* ≈ 0.5 nm^–1^.^65^

There is an observable decrease in intensity from the dry to the wet profile due to the presence of liquid water. The parallel shift of intensity (intensity decrease without changing of the slope corresponding to a change of contrast) indicates that the wetting is homogeneous at all length scales. Porosity determined by the invariant integration of absolute scaled intensity within 0.02 < *q* < 0.8 nm^–1^ (excluding *q* range where the intensity saturated the detector *^1^ and Mylar background from beam flight tube *^2^) was ≈96.2 ± 0.3%. The standard error of mean was obtained from the error value of the averaged 100 SAXS profiles in different locations. The large discrepancy to the manufacturer porosity (≈70%) may be explained by the limited experimental *q*-range and the nature of the material. To obtain an accurate invariant value, the measured SAXS intensity should approach a constant value at low *q*, which is not the case for the PVDF sample (see Figure 3a and Kratky plot in Figure S3a).The liquid-water saturation level determined by the invariant integration was ≈100% by using the porosity value provided by the manufacturer. Given PVDF’s similar SLD as the carbon’s (SLD_PVDF_ = 15.15 × 10^10^ cm^–2^ versus SLD_carbon_ = 17.82 × 10^10^ cm^–2^), we concluded that with the presence of liquid water, an intensity change should be observable also in the CL.

### Ex Situ Wetting of Non-noble Metal Catalyst Layer by n-decane

To assess the detectability of liquid water in the CL, a two-phase (solid-void) non-noble metal CL was wetted by *n*-decane, a highly wetting, model liquid with low evaporability at room temperature (0.17 kPa vapor pressure at 25 °C). The advantage of using a non-noble metal CL in terms of data analysis is that there is no regard to Pt contribution needed as it is Pt-free. The SAXS 1D intensity profiles of the NNMC CL in the dry state vs wetted by *n*-decane show that a power law decay with an intensity of I(*q*) ∼ *q*^–3.^(37) for both the dry and wetted sample (see Figure 3b, Kratky plot in Figure S3b). The exponent of −3.37 covering a *q*-range larger than one decade indicates a surface fractal behavior, in which the sample’s surface structure is rough.

The intensity shift from dry to wet is homogeneous over the whole *q*-range similar to the case of the hydrophilic porous membrane wetted by water (vide supra). The calculated porosity is ≈90.7 ± 0.6% (from invariant integration of the dry profile in absolute unit versus ≈86% from thickness measurement) and ≈102% liquid-saturated from the wet profile using the porosity from thickness measurement. The very high porosity may be attributed to contribution of pores within the catalyst aggregates. It may be also due to the insufficient measurement *q*-range as the intensity at low-*q* is only starting to plateau, meaning that the pore sizes examined may be broader than the accessible *q*-range.

### Ex Situ Wetting of Pt/C by *n*-decane

The detectability of the liquid water present in the CLs is further assessed using the model liquid *n*-decane and the Pt/CCL. A SAXS 1D profile of the Pt/C CL wetted by *n*-decane is shown in Figure 3c. In the lower-*q* region (0.015 < *q* < 0.08 nm^–1^), a power law decay with I(*q*) ∼ *q*^–1.69^ for the dry and *I*(*q*) ∼ *q*^–1^(54) for the wet sample is observed. In the intermediate region (0.08 < *q* < 0.15 nm^–1^), different power laws are observed, *I*(*q*) ∼ *q*^–3^(42) for the dry and *I*(*q*) ∼ *q*^–3^(34) for the wet state. There is a bump in the high-*q* region (0.6 < *q* < 1 nm^–1^) that is attributed to the Pt nanoparticles with particle sizes of ≈ 2–3 nm.^33^ The deviation from I ∼ *q*^–4^ indicates that there may be a fractal behavior or polydisperse particle size. The exponent of > −2 (≈−1.7) covering about a decade of *q* in the low-*q* region is due to mass fractal behavior, and exponents > −4 (≈−3.4, covering only less than a decade of *q* due to the Pt bump in the intermediate *q*-region) may be due to the surface fractals (i.e., self-similar rough surface structures).^66^

The intensity shift from the dry to the wetted sample is inhomogeneous over the entire *q* range and more pronounced at lower *q*. This is attributed to the constant contribution of Pt which dominates at high *q* (SLD_Pt_ is ≈10x higher than of the other components). The porosity was determined as ≈ 53.4 ± 10% (assuming carbon density of 2.1 g/cm^3^, 62% porosity with 2.2 g/cm^3^) by the dry structure invariant integration of absolute unit scaled intensity compared to ≈52% from sole thickness measurement. The accurate invariant analysis of the Pt/C sample can be explained by the fact that the invariant integration with the given experimental *q*-range approximates the integration from *q* = 0 to *q* = ∞ reasonably well. Much more pronounced compared to PVDF and NNMC, the SAXS intensity approaches a constant value at low *q* (see Figure 3). The Kratky plot in Figure S3c further supports this. At *q* near 0, *I*(*q*) × *q*^2^ approaches 0, as opposed to PVDF and NNMC. Based on the invariant calculation using the porosity from thickness measurement (see the Materials in the Experimental section), the saturation level by *n*-decane was estimated as ≈ 101%, assuming a constant Pt contribution.

### Dry Structure Morphological Modeling

Now that the detectability of liquid in the pores of the porous membrane and the CLs has been confirmed and the behavior of fully wetted SAXS intensity profiles was characterized, a relationship between the changes in the SAXS profiles and the wetting mechanism needs to be established. It is important to note again that the invariant calculation used above could give quantification of the saturation level. However, for a *q*-dependent pore-filling mechanism given a saturation number, a structural model needs to be constructed.

**Figure 4:**
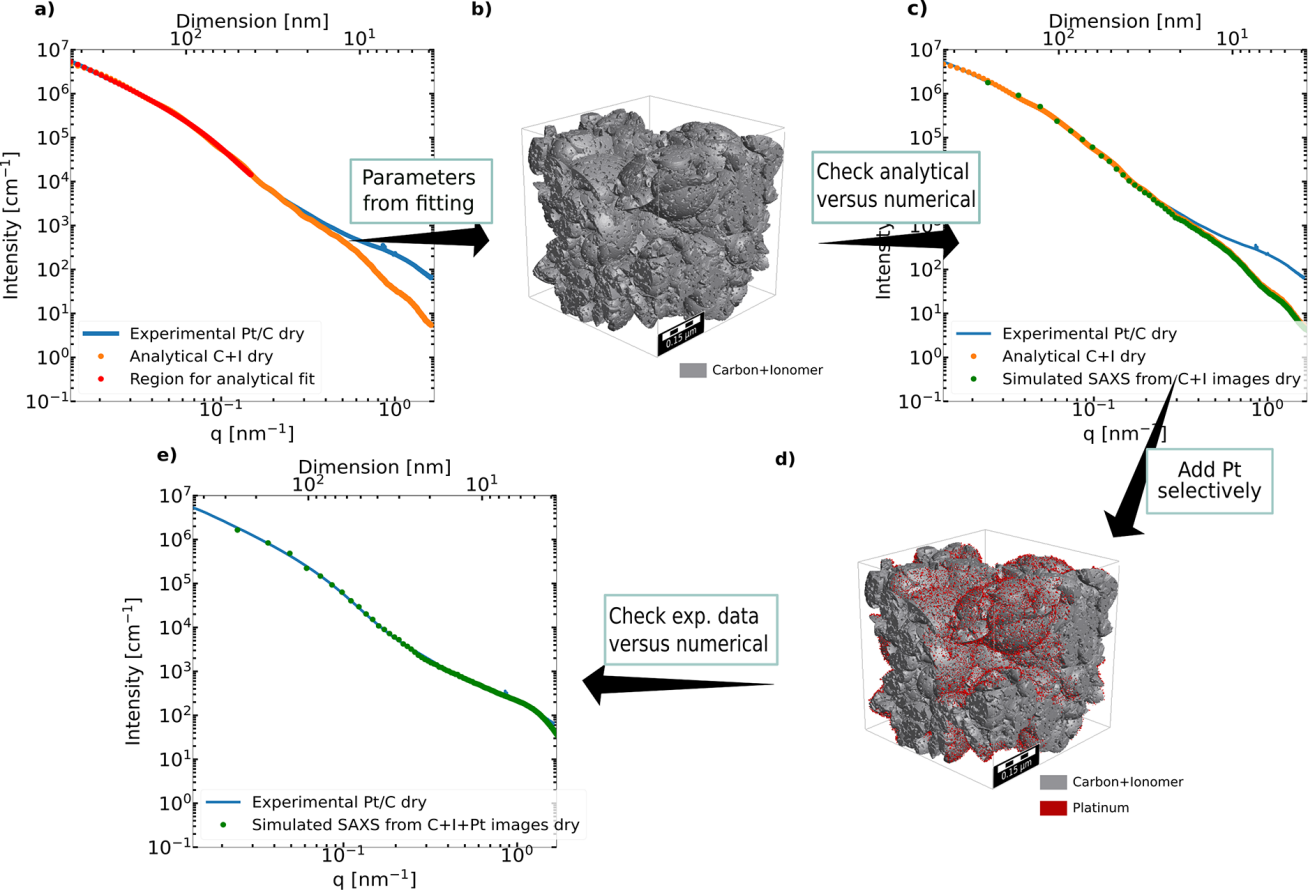
(a–e) Flowchart of the process followed for developing a morphologically representative model for a CL. (a) Fitting the low-q region of the SAXS profile with the analytical solution of the IBM. (b) 3D representation generated with the fitting parameters. (c) Analytical solution versus simulated scattering from IMG2SAS. (d) Selectively adding Pt nanoparticles to the structure. (e) Final fit of the experimental data with simulated scattering; top axis is real space dimension D corresponding to 2π/*q*.

Figure 4 shows a flowchart of dry Pt/C structure generation, which involved a choice in the appropriate model. The Pt/C CL is known to consist of aggregates and agglomerates of primary carbon particles, resulting in a fractal structure.^21^ To accommodate the fractal nature of the carbon support in Pt/C and the existence of hierarchical pores, the IBM was chosen, see the section SAXS Data Interpretation Tools.^42,46^ The model SAXS intensity of the IBM assumes a porosity of 52% (obtained from thickness measurement of CL, see the Experimental section) and was fitted (eq 4 is minimized) in the low-*q* region of the Pt/C CL experimental data (red dots) in Figure 4a, which corresponds mainly to the contribution from the support. The obtained parameters are then used to generate a 3D representation of the carbon + ionomer structure (see Figure 4b). The same parameters could generate many 3D structures; therefore, the presented structure is not unique. Nevertheless, the statistical correctness of the generated structure is validated by the good agreement between the corresponding SAXS simulated intensity and the analytical model fit and the experimental data (for *q* ≤ 0.3 nm^–1^) (see Figure 4c). The discrepancy in the high-*q* region comes from the scattering of Pt. By putting randomly distributed Pt particles (0.6% vol.) on the surface of the carbon + ionomer solid,^67^ the Pt/C scattering can be matched over the entire available *q*-range (Figure 4d shows the visualization of the resulting scattering curve 4e) (see the SAXS Data Interpretation Tools section d).

**Figure 5:**
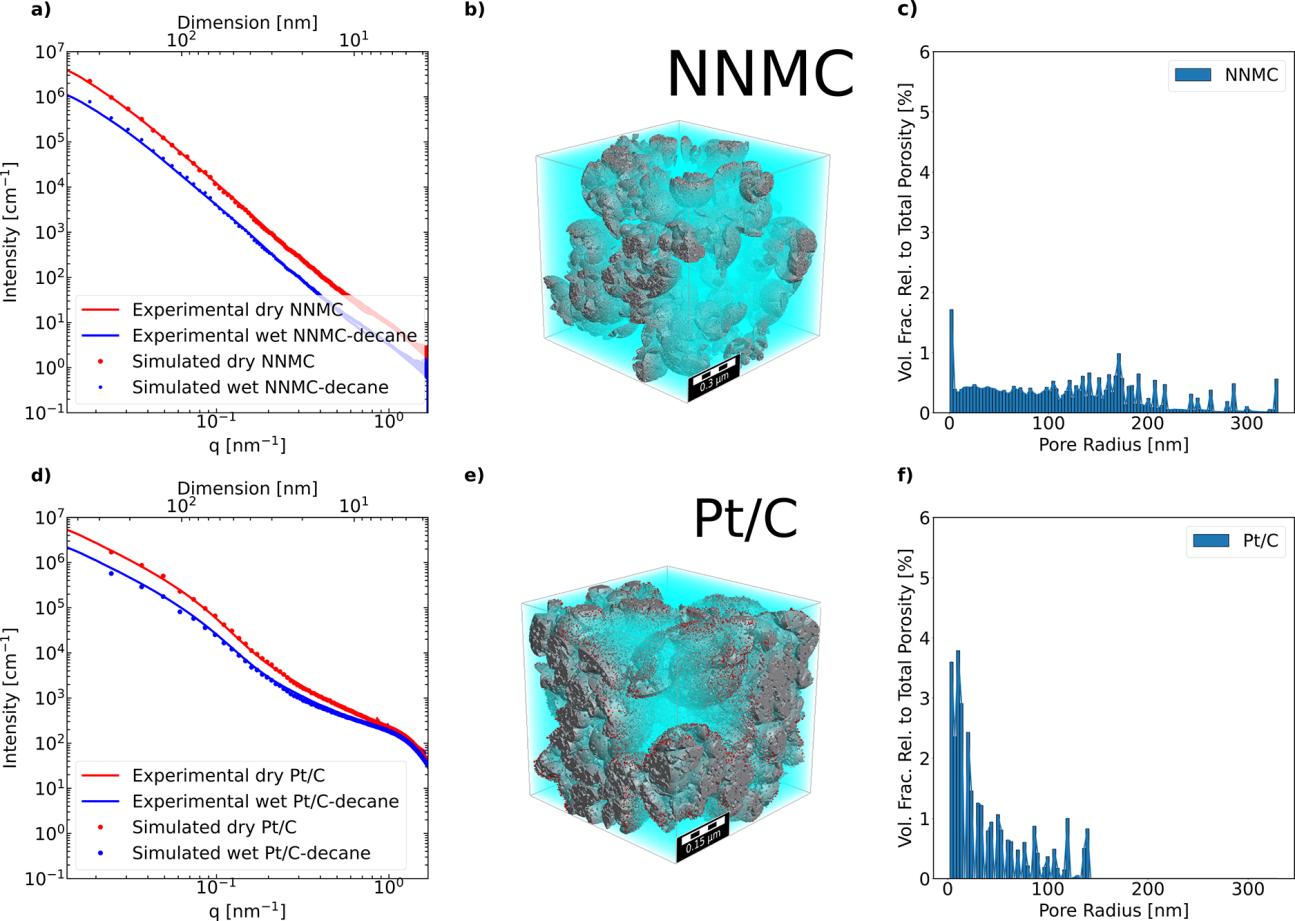
Experimental-simulation fit for NNMC-decane and Pt/C-decane (a,d); top axis is real space dimension D corresponding to 2π/*q*. Resulting structure corresponding to the simulated SAXS profile (b,e). Pore size distribution of the 3D representation (c,f).

It is important to note that the process of placing Pt on the Pt/C catalyst structure also took into account the fully wet data to gain more confidence about the representativeness of the fit by minimizing using the error function in eq 5. In the case of the NNMC, fitting the SAXS intensity of the dry is sufficient to have well matched dry and wet (see Figure 5a–c). Figure 5d–f shows a good match between the experimental and modeled SAXS intensities of Pt/C in dry and *n*-decane wet state. The pore size distribution of the simulated structure was calculated using the granulometry module of Geodict2021 (Math2Market GmbH, Germany). The results confirm the existence of both expected, sub-10 nm primary pores and 10–150 nm secondary pores for a Vulcan XC72-based Pt/C catalyst (see Figure 5f).^68^ The pores of NNMC are bigger than the pores of Pt/C from the pore size distribution of the generated structure, also reflected by the intensity levelling off at low *q* happening at lower *q* than for the Pt/C structure. The IBM fitting parameters from analytical fits for both NNMC and Pt/C, as well as the Pt amount and placement fit parameters are compiled in Table 2 (for further details about IBM, see the Supporting Information). Since there can be only solid carbon on locations where spheres of all classes overlap, the pores show up where at least one class of spheres is not represented. In the structures generated in this work, the pores are mainly introduced by the smaller classes of IBM into the final structure, but this must not hold true in all cases as the morphology of the solid matrix and the pores depend on the solid volume fraction parameter of all classes of spheres. Due to the higher sub 10 nm porosity of the NNMC electrodes, indeed smaller IBM-radii classes are required to match the scattering data compared to the LSC-Vulcan Pt/C electrodes. For the Ketjen-Black-based Pt/C electrode with higher sub 10 nm porosity, smaller IBM-class radii also would be required to properly reconstruct the SAXS data. Chord length and two-point function analysis of both CLs are given in Figures S21 and S22 of the Supporting Information. Experimental or imaging-based verification of the reconstructed structures should be aimed at in future work, though covering the wide feature size range of CLs and matching the sensitivity range of SAXS remain a challenge in itself and may require a combination, i.e., MIP, (FIB/)SEM, TEM, and nano-CT.

**Table 2:** Parameters from the Experimental-Simulated Scattering Fit for NNMC and Pt/C^a^

	NNMC		Pt/C	
IBM Class(i) [−]	*R*_*i*_ [nm]	ϕ_1,*i*_ [ %]	*R*_*i*_ [nm]	ϕ_1,*i*_ [%]
1	0.5	90.1	5	96.2
2	2.5	95.3	26	95.5
3	3	95.6	47	96.3
4	6	92	60	93
5	9	95.2	114	95.2
6	42	97	125	93
7	78	96.7	159	90.5
8	92	93.6	190	92.9
9	104	98.2	0	0
10	178	98.4	0	0
Pt1	not applicable	not applicable	2	0.52
Pt2	not applicable	not applicable	3	0.1

a For each IBM class, there exists corresponding radius of Boolean spheres *R*_*i*_ and the solid volume fraction ϕ_1,*i*_ (or 1-ϕ_0,*i*_). Additionally for Pt/C, there are free parameters from Pt nanoparticles consisting R and its volume fraction *V*.

### In Silico Wetting Scenarios and Their Relation to the SAXS Profiles

Taking advantage of the statistically representative 3D solid CL structure, we further investigate the influence of the wetting mechanism on the SAXS profiles. The mechanisms investigated are (I) small-pores-filling-first, (II) large-pores-filling-first, and (III) thin-film-formation of water. More complex models (for example, nucleation site of water growing from Pt, surface energy minimization based on contact angle) could be explored as well but there is a tradeoff between model complexity and its computational cost. Filling artificial water to a generated structure with morphological operation and subsequent conversion to the SAXS profile roughly takes 20 s which is already relatively long for fitting procedures where hundreds/thousands of iterations are expected.

**Figure 6:**
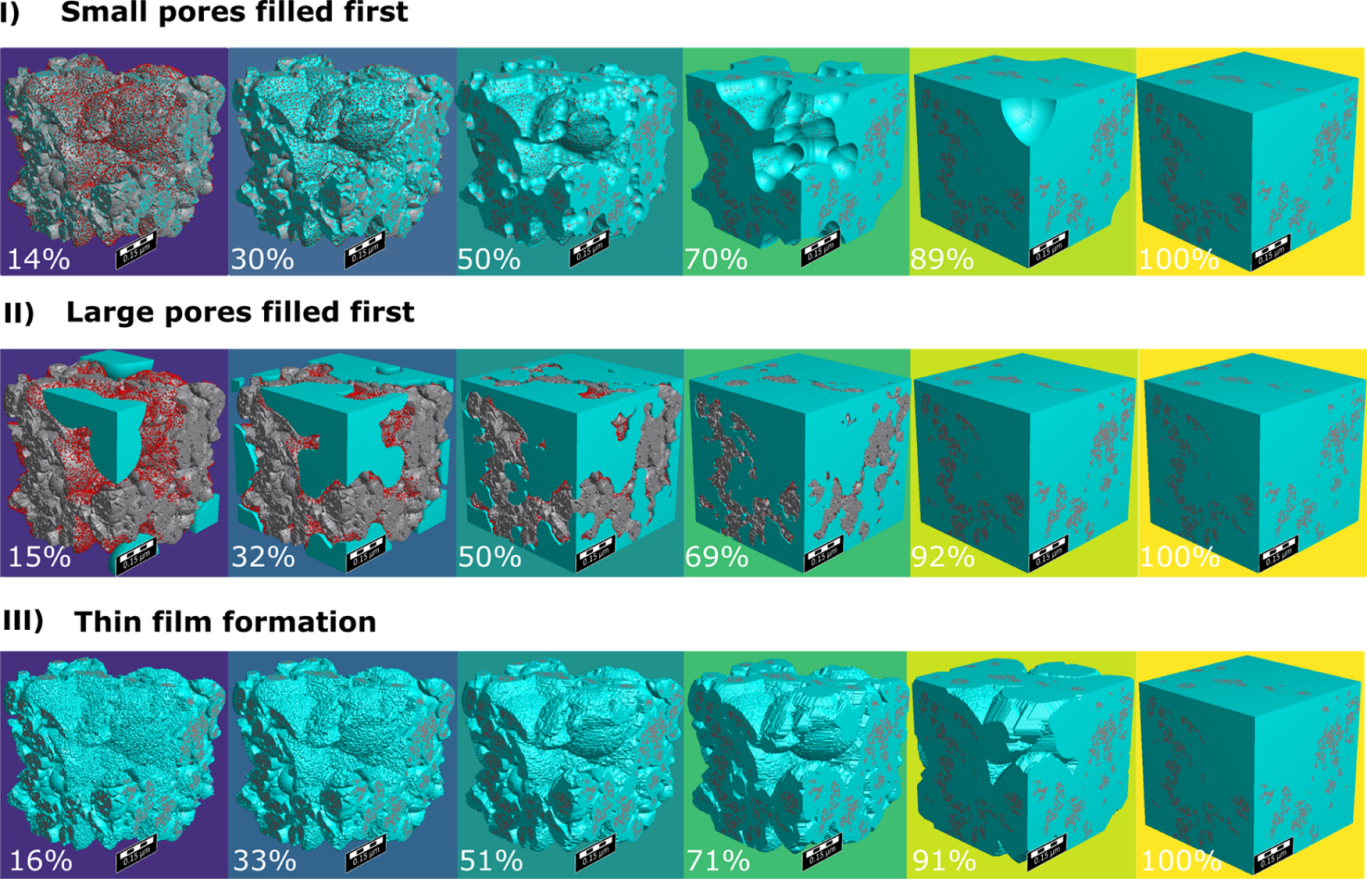
Different mechanisms of wetting in the pores of Pt/C: (I) smaller pores filling first, (II) larger pores filling first, and (III) thin-film formation. The corresponding water saturation levels are indicated as percentage. Scale bar is 0.15 μm.

**Figure 7:**
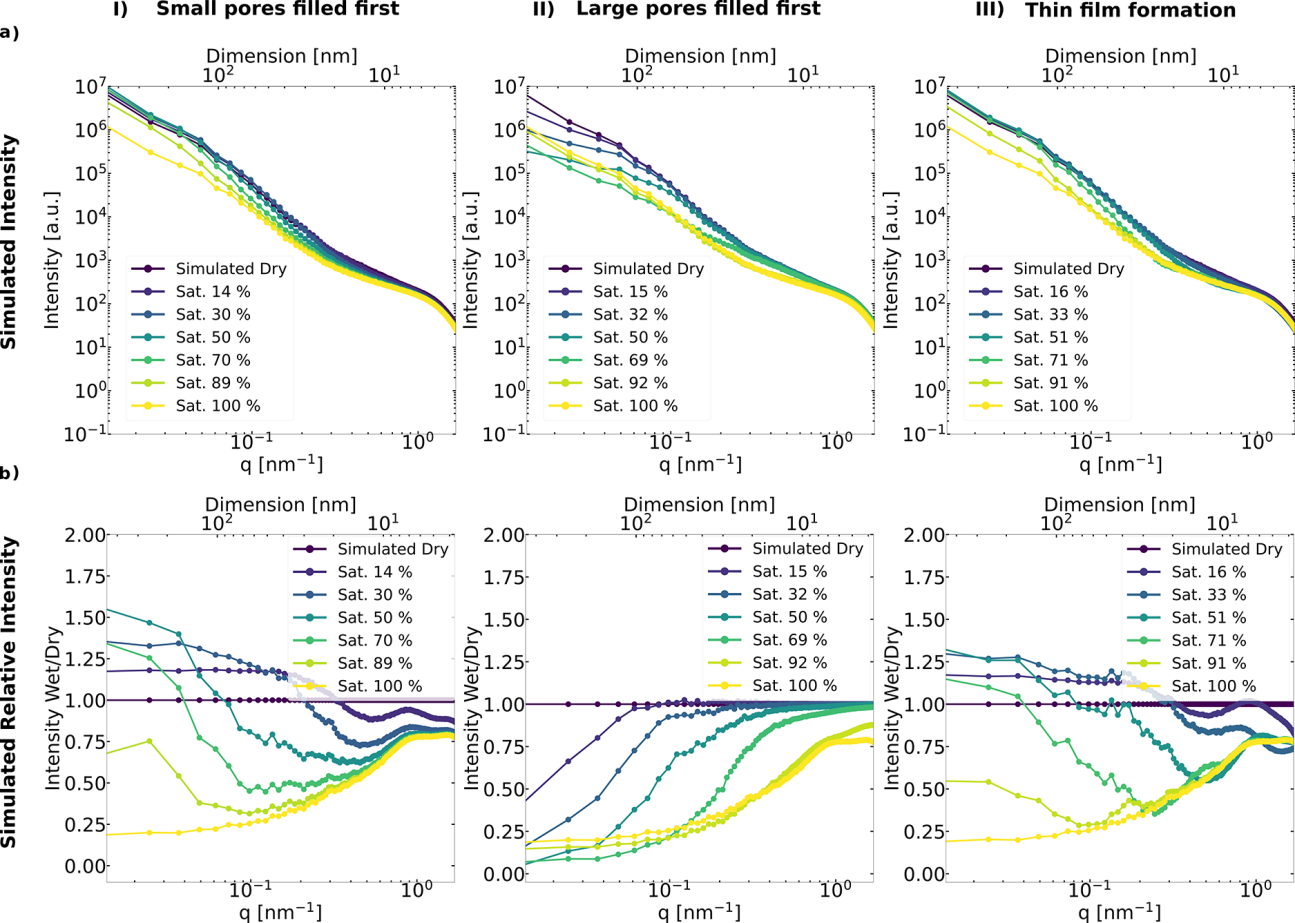
Simulated SAXS profiles of Pt/C (a-I-III) with different saturation levels in the three wetting mechanisms explored, and its corresponding relative intensities (wet/dry) to visualize better the intensity change (b-I-III). The curves are color coded similarly to Figure 6; top axis is real space dimension D corresponding to 2π/*q*.

The structural representations of each mechanism are shown in Figure 6I–III, respectively. Similarly color coded as the 3D structure, the simulated 1D SAXS profiles of these structures are shown in Figure 7a. The relative intensity (intensity wet/dry) is plotted in Figure 7b to better visualize the *q*-dependent intensity changes upon wetting. Water saturation levels in the legend are deduced from the sum of water-filled voxels in each structure.

When small pores are filled, a change in contrast from void to water results in a decrease of scattering intensity initially observed mainly in the intermediate-*q* (0.1 < *q* < 1 nm^–1^) and high-*q* region (*q* > 1 nm^–1^). As water fills small pores, they create larger structures with higher mean SLD, increasing the scattering at lower-*q*. Then, as it fully fills, intensity at low-*q* decreases.

In the case of the large-pores-filling-first scenario, an intensity decrease is observed initially in the low-*q* (*q* < 0.1 nm^–1^) region that spreads toward higher-*q* as the saturation increases. Only at very high saturation levels, beyond 90% changes of the scattering intensity at very large *q* > 1 nm^–1^ can be observed. The smallest pores fill only at the very end under these conditions.

For the thin-film water formation scenario, the changes in scattering intensity are similar to the case in which small pores fill first. The intensity primarily decreases at higher *q* because the small real space distance of a thin film is indeed similar to the small-pores-first scenario. A film of finite thickness on the entire carbon/ionomer surface will automatically fill the small pores first. The initial increase at low *q* is less pronounced, whereas thin-film formation leads to a narrower peak at large *q*. The thin-film water formation scenario assumes a homogeneous thickness of water film formation. In reality, the thin film of liquid water may depend on the ionomer coverage on the carbon/Pt surface, which is not necessarily homogeneous. Therefore, to model inhomogeneity of water thin film, an additional water film “roughness” parameter may be incorporated in future studies.

As expected for Pt/C, due to the high electron density of Pt, the intensity change between fully wetted and dry sample is not uniform over the entire *q*-range. For the Pt-free NNMC CL (derived from the Pt/C structure without Pt), the trends are similar under partially wetted conditions, as shown in Figures S5 and S6.

### Examination of Pt Contribution to Saturation Levels Obtained from SAXS Invariants

**Figure 8:**
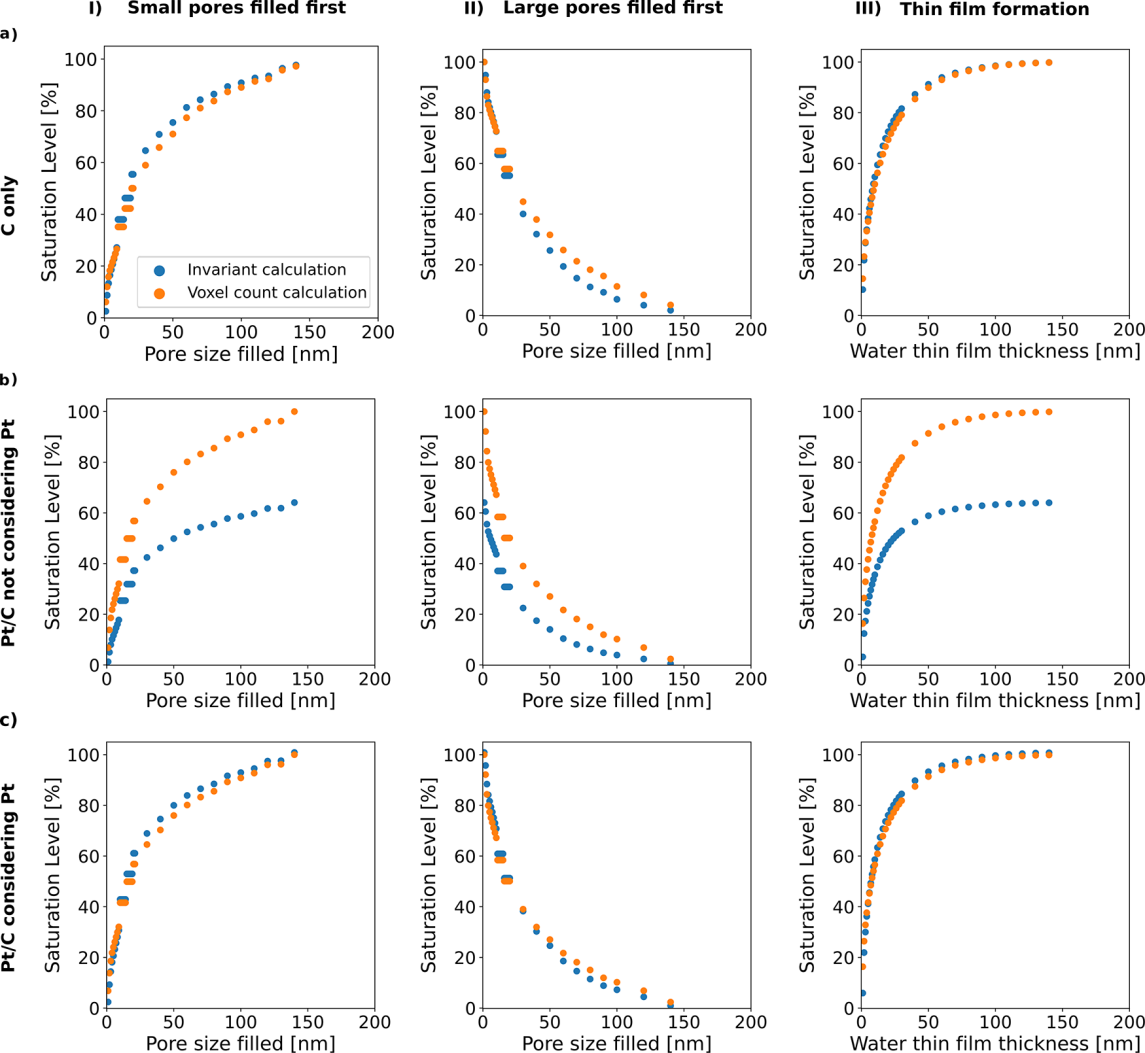
Saturation level from invariant calculation of simulated scattering versus voxel counting for (a) carbon only, (b) Pt without accounting for Pt contribution, and (c) Pt/C with contribution Pt. I is from small pores filling first, II is from large pores filing first, and III is from the thin-film formation wetting mechanism. Pore size is pore radius-based.

In order to further validate the significance of the Pt contribution to the saturation level calculation, the invariant-based saturation level calculation is not only applied for the experimental data but also to the simulated, partially wetted 3D structures and their corresponding SAXS intensities. The saturation level obtained from the invariant calculation of the Pt/C structure without the Pt or carbon only for the three wetting mechanisms is close to the saturation level obtained by voxel counting (Figure 8a). The two-phase assumption (solid-void) was applied to calculate the saturation level of Pt/C (see Figure 8b), where a huge discrepancy of up to 40% and an underestimation of the saturation are observed compared to the saturation level by water voxel counting.

Subtracting the Pt scattering contribution (see Methods section) results in saturation levels close to the ones obtained by voxel counting. This works for the Pt/C CL for all three filling scenarios over a wide saturation range (see Figure 8c). In general, the estimated discrepancy between the saturation level from the invariant calculation and from voxel counting is within 5% for the Pt/C CLs.

### In **Situ Wetting of Pt/C**

**Figure 9:**
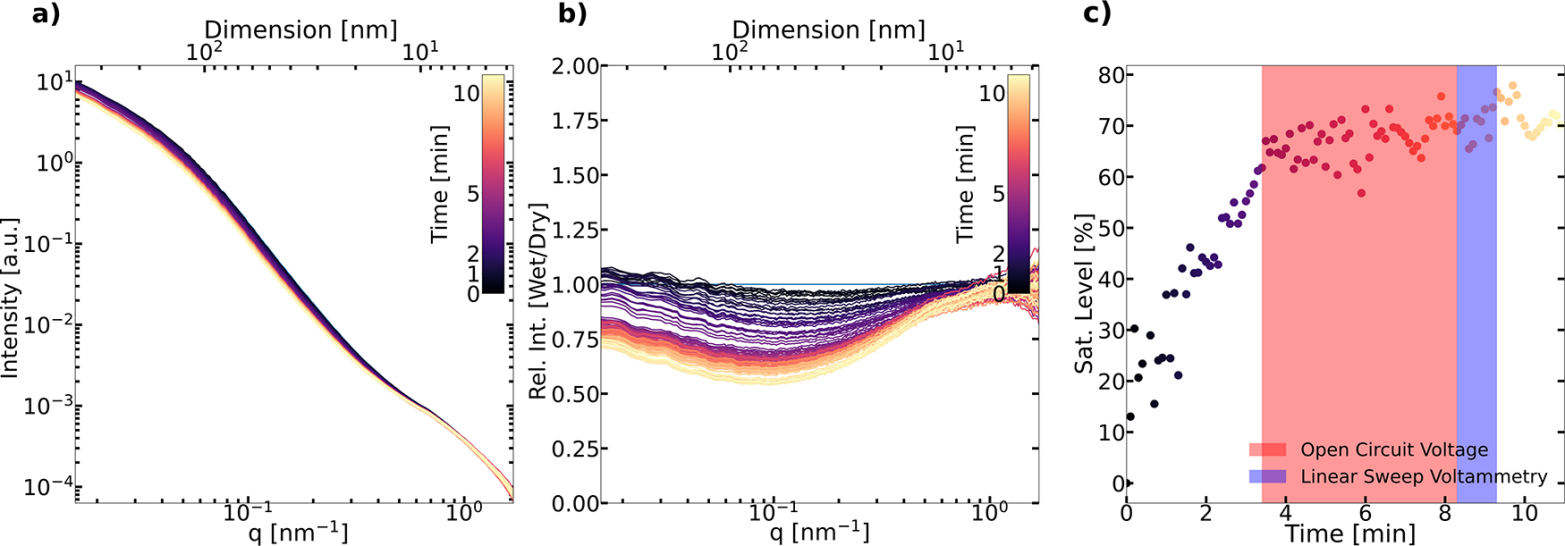
(a) SAXS profiles taken during in situ wetting of the Pt/C CL within the first 10 min, (b) relative intensity (wet/dry) of the SAXS profile over time, and (c) saturation level from the SAXS profile versus time.

Based on the presented methodology to interpret SAXS data and quantify liquid water saturation in Pt/C CLs, time-resolved in situ experimental data from fully dry to wet states in an in situ flow cell are analyzed next. The time-resolved SAXS data show a decrease in intensity at 0.02 < *q* < 1 nm^–1^ and have little change at *q* > 1 nm^–1^ (Figure 9a,b). The saturation levels were calculated using the SAXS invariant calculation approach on the time-dependent scattering curves after removal of Pt contribution. The saturation level increased sharply to a saturation level of ≈65% within 3 min, plateauing thereafter at ≈ 70% (Figure 9c). The saturation uncertainty due to the applied carbon density (2–2.2 g/cm^3^) to saturation calculation is ≈ 4%. Radiation-induced changes to the structure are negligible for a few SAXS measurements and may contribute max 7% to saturation changes even after 2x higher accumulated X-ray dose (for details, see the Supporting Information).

From the relative intensity plot in Figure 9b, an intensity decrease is observed at 0.1 < *q* < 1 nm^–1^ followed by a gradual decrease in intensity toward low-*q*. The trend is consistent with both the small-pores-filling-first and the thin-film formation scenarios above. A rather flat relative intensity in the *q* range 0.1 < *q* < 1 nm^–1^ is observed in Figure 7bI at a saturation level of 50% and is observed in the experimental data in Figure 9b but is not present in the thin-film formation model. For both the small pores filling first and the thin-film formation scenario, an intensity increase at the low-*q* (*q* < 0.1 nm^–1^) is expected (as discussed above, see Figure 7bI,III). However, the experimental data show that only a small intensity increase at lower *q* can be observed (Figure 9). We believe that this behavior may indicate that the expected intensity increase is counterbalanced by a simultaneous significant fraction of larger pores being filled. We conclude that the most likely wetting mechanism is a combination of the large pores filled and the small pores (20–30 nm) filled first scenarios.

This seems reasonable considering the capillary forces if the catalyst + ionomer is rather hydrophilic. In the in situ experiments, the electrolyte wets the CL only on one side. There could be an impregnation of the CL from the side in contact with the electrolyte toward the side in contact with the Kapton film. Thus, the evolution of saturation and type of wetted pores might not be homogeneous over the thickness of the electrode during the duration of the experiment, while the collected SAXS data provide only thickness averaged data. It could be that the larger pores in the CL close to the electrolyte are filled so that the smaller pores in the CL in contact with the Kapton film can be filled.

Additionally, and in agreement with the literature, changes in the Pt particle size (from ≈2.2 to ≈2.6 nm, also growing and shrinking periodically) were observed during the cycling of the flow cell electrode (see Supporting Information Figure S8).^69^

## Conclusions

This paper presents SAXS as a suitable methodology to quantify the liquid saturation in a multiphasic CL system. The methodology has been applied to model systems and a conventional Pt/C in ex situ and in situ measurements. First, the detectability of liquids in various CLs has been demonstrated, and it was shown that liquid saturation can be quantified using SAXS. Second, in silico experiments and stochastic modeling were used to interpret the complex multiphase by generating statistically representative 3D real-space structures of IBMs. For Pt/C CLs with large contributions of Pt to the measured SAXS profiles due to its high electron density, a careful subtraction of the contribution of Pt is needed to obtain the true water saturation level.

Additionally, there could exist different filling modes that yield the same saturation level. In silico SAXS intensity profiles have been shown in this study to capture the difference coming from pore-filling modes, specifically small-pores-filled-first, large-pores-filled-first, and thin-film-formation. Third, the in situ wetting behavior of a Pt/C CL has been investigated where we observed a tendency for small pores filling first in combination with a fraction of the large pores being filled concluding from the in silico filling results.

In the future, in silico experiments that allow for a combination of the filling modes or incorporating even more physics-based models considering nucleation sites or surface minimization need to be developed. This could enable a more precise model fit and the quantification of pore size and volume fraction of the filled pores. Even though the filling of the working electrode with the liquid electrolyte in the flow cell does most likely not match the wetting mechanism in a CL in an operating PEFC one to one, the presented SAXS data analysis tools and approach are however foreseen to enable the determination of the water saturation level and the water wetting mechanism in CLs, given the development of an appropriate SAXS-compatible operando setup in the future. This, in turn, will close the gap of currently available diagnostic methods that could not provide insights into pore size-specific saturation in the CL under operando conditions on a wide nanometer scale and will enable advancements in CL material design.

## Supporting Information Available

Details on invariant calculation IMG2SAS; analytical solution for the representative morphological model; image generation of the representative morphological model; water simulation on the representative morphological model; and form factors of various shapes and other morphological model with IMG2SAS (PDF)
