# Supplementary material for: Quantification of PEFC Catalyst Layer Saturation via In Silico, Ex Situ, and In Situ Small-Angle X-ray Scattering
Source: ACS Appl Mater Interfaces. 2023 May 25;15(22):26538–53. doi: 10.1021/acsami.3c00420 (PMC10251350; doi:10.1021/acsami.3c00420)
Supplement: Supplementary file 1 — am3c00420_si_001.pdf [file am3c00420_si_001.pdf]

Supporting Information

Quantification of PEFC Catalyst Layer Saturation via *In silico*, *Ex* and *In situ* Small Angle *X*-ray Scattering

*Kinanti Aliyah[1], Christian Prehal[2], Justus S. Diercks[1], Nataša Diklić[1], Linfeng Xu[1], Seçil Ünsal[1], Christian Appel[3], Brian R. Pauw[4], Glen J. Smales[4], Manuel Guizar-Sicairos[3], Juan Herranz[1], Lorenz Gubler[1], Felix N. Büchi[1], Jens Eller[1]**

Affiliation(s):

[1] Electrochemistry Laboratory, Paul Scherrer Institut, 5232 Villigen PSI, Switzerland

[2] Department of Information Technology and Electrical Engineering, ETH Zürich, 8092 Zürich, Switzerland

[3] Photon Science Division, Paul Scherrer Institut, 5232 Villigen PSI,

Switzerland

[4] Federal Institute for Materials Research and Testing (BAM), 12205 Berlin, Germany

*Corresponding Author

Jens Eller [jens.eller@psi.ch]

Experimental Setup for *Ex situ* and *In situ* Wetting

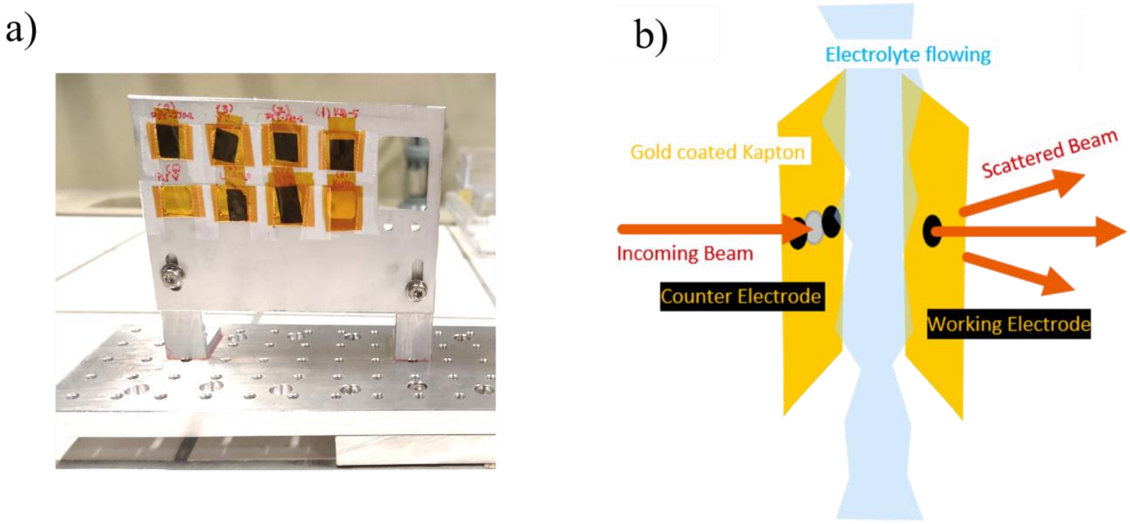

Figure S1 a) Sample environment of ex situ wetting. Samples are mounted perpendicular to the beam and are placed in Kapton bags to prevent liquid evaporation. b) Scheme of in situ wetting experiment. Catalyst layer of interest is coated on a conductive Kapton here denoted as working electrode. A more details setup information can be found in [1].

Subtraction of Pt constant contribution

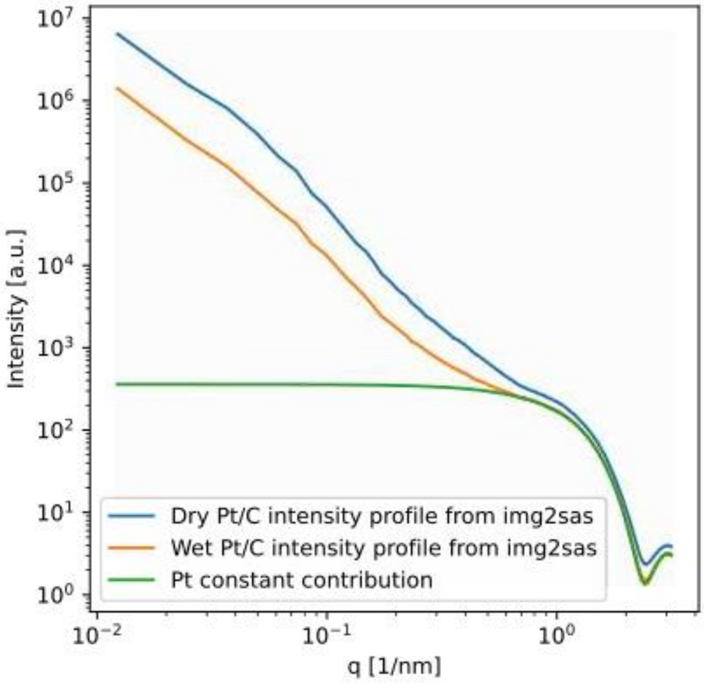

Figure S2 Intensity profiles of dry and wet Pt/C from 3D simulated structure and Pt constant contribution for calculating the Invariant.

Kratky Plots of *ex situ* wetting samples

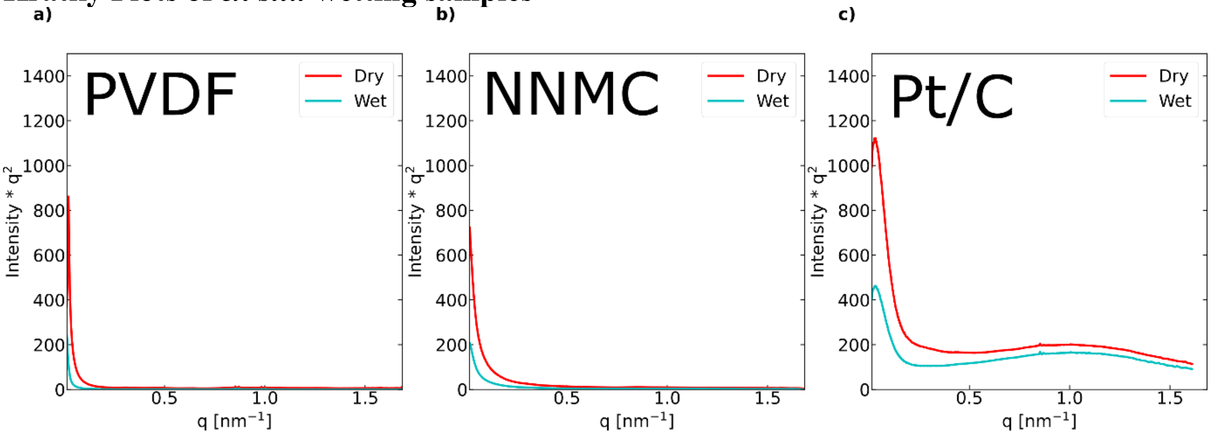

Figure S3 Kratky plots (I(q) * q ^2) versus q for a) PVDF, b) NNMC, c) Pt/C. Among the three, only Pt/C catalyst layer has intensity down turn at q near 0, indicating sufficient q-range from the experimental setup.

Determination of the liquid saturation level by Invariant calculation

Invariant is related to the electron density and the volume fraction of the material by the following equation expressed for 3-phase system.

$$\frac{\tilde{I}}{2\pi^2} = K((\rho_A - \rho_B)(\rho_A - \rho_C)[\phi_A - \phi_A^2] + (\rho_B - \rho_A)(\rho_B - \rho_C)[\phi_B - \phi_B^2] + (\rho_C - \rho_A)(\rho_C - \rho_B)[\phi_C - \phi_C^2]) \quad \textbf{(Equation 1)}$$

Where ρ_i is the scattering length density (SLD) of the material *i* and ϕ_i the volume fraction of the material *i* and K is scaling factor from the integrated experimental data (left side) to the invariant calculated by the formula (right side).

The volume fraction of liquid inside a material is deduced by first obtaining the experimental constant *K* scaling the theoretical value of Invariant given a porosity/volume fraction of the material, to the Invariant from dry experimental data. For the dry case, which is 2-phase, the Invariant calculation is done by keeping only the A (as solid) and B (as void) phases and setting ρ_c = 0 and ϕ_C = 0 , also ρ_B =0 due to the density of void being 0 in Equation 1. Equation 1 is then reduced to Equation 2 where K is solved by putting in the integrated intensity of the dry structure and scattering length density of the solid. A-priori knowledge of the porosity and density of the material in dry structure is needed for this calculation.

$$\frac{\tilde{I}}{2\pi^2} = K\left((\rho_A{}^2)[\phi_A - \phi_A^2]\right) \quad \textbf{(Equation 2)}$$

The value of K obtained from Equation 2 was then use in Equation 1 to solve the ϕ_C or volume fraction of liquid (see Equation 3).

$$\phi_c = \frac{-b \pm \sqrt{b^2-4ac}}{2a} \quad \textbf{(Equation 3)}$$

, where $a = \left(-(\rho_C - \rho_A)(\rho_C - \rho_B) - (\rho_B - \rho_A)(\rho_B - \rho_C)\right)$,

$$b = \left(-(\rho_B - \rho_A)(\rho_B - \rho_C) + \left(2(1-\phi_A)(\rho_B - \rho_A)(\rho_B - \rho_C)\right) + (\rho_C - \rho_A)(\rho_C - \rho_B)\right),$$

$$c = (\rho_A - \rho_B)(\rho_A - \rho_C)[\phi_A - \phi_A^2] + (\rho_B - \rho_A)(\rho_B - \rho_C)(1-\phi_A) - ((1-\phi_A)^2 - \frac{\frac{\tilde{I}_{wet}}{2\pi^2}}{\frac{\frac{\tilde{I}_{dry}}{2\pi^2}}{((\rho_A{}^2)[\phi_A - \phi_A^2])}}.$$

In the case of absolute-scaled intensity of a two-phase material can be experimentally obtained, K in Equation 2 equals to 1 (experimental Invariant equals to theoretical Invariant). The porosity (1-solid fraction ϕ_A) can be calculated if the SLD (ρ_A) of the solid fraction in void is known.

A SLD of hydrophilic porous PVDF membrane 15.15x10^{10} cm^{-2} and porosity of 70% were used. The SLD of the dry catalyst layer structure is the volume-weighted average of the SLD of ionomer and carbon, which are 17.82x10^{10} cm^{-2} and 16.36x10^{10} cm^{-2}. The SLD of water is 9.45 x10^{10} cm^{-2} and of decane is 7.16 x10^{10} cm^{-2}. All SLDs are calculated based on 11.2 keV beam energy using Equation 4, where Z is the atomic number of the i th element in the molecular volume v_m and r_e = 2.81 X 10^{-13} cm, is the classical radius of the electron.

$$\mathrm{SLD} = \frac{\sum_{i=1}^{n} \mathrm{Z} r_e}{\mathrm{v_m}}$$ **(Equation 4)**

Details on Intersected Boolean Model

The geometrical covariogram of randomly distributed spherical grains i reads

$$K_i(r) = \frac{4\pi}{3} R_i{}^3 \left(1 - \frac{r}{2R_i}\right)^2 \left(1 - \frac{r}{4R_i}\right) \Theta(2R_i - r)$$ **(Equation 5)**

where r is the real space coordinate, and Θ() the Heaviside step function, being 1 for a positive argument and 0 otherwise. The solid covariance C11(r) is the probability that a stick with length r, with random position and direction, has both of its ends in the solid phase of the two-phase pore structure. It can be calculated from the geometrical covariogram $K_i(r)$. With N being the class of the Boolean model

$C_{11}(r) = \prod_{i=1}^{N} \phi_{0,i}^2(\exp[\theta_i K_i(r)] - 1) + (1 - \phi_{0,i})^2$ (**Equation 6**)

$\phi_{0,i}$ is the pore volume fraction of a specific class of grains i and equals $\exp(-\theta_i 4/3\pi R_i^3)$ where θ_i is the number density of grains in the simulation box. The number density of grains θ_i is adjusted such that $\phi_1 = \prod_{i=1}^{N}(1 - \phi_{0,i})$ equals the total volume fraction of the solid phase. The model scattering intensity I(q) is obtained by numerically integrating.

$I(q) = K \cdot \phi_1(1 - \phi_1)(\Delta\rho)^2 \int_0^\infty \gamma(r) \frac{\sin(qr)}{qr} 4\pi r^2 dr$, with $\gamma(r) = \frac{C_{11}(r) - \phi_1^2}{\phi_1(1-\phi_1)}$ (**Equation 7**)

This model scattering intensity is fitted to the measured intensity. The grain sizes R_i, the number density of grains θ_i, and the prefactor K are constant during the experiment and are solved as fitting parameters.

Selective Pt placement on Carbon+Ionomer Structure

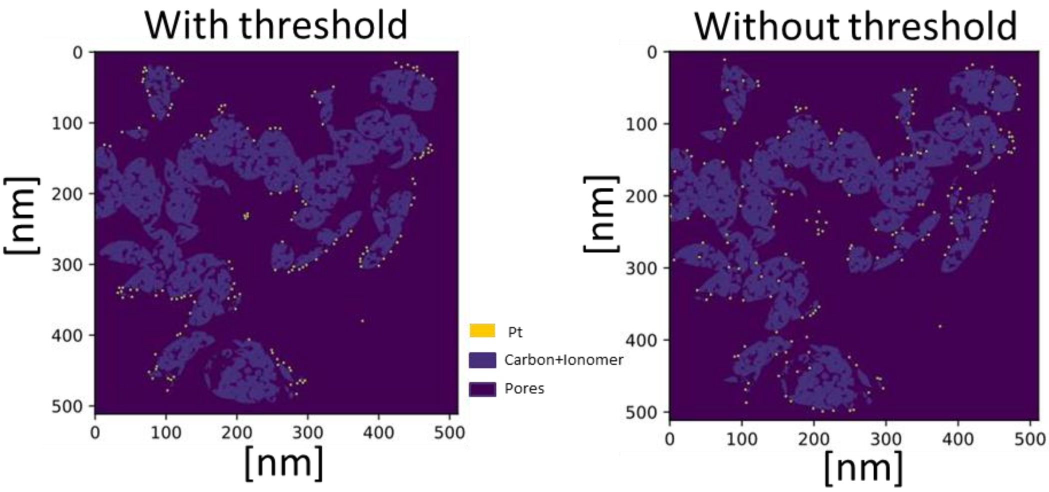

Figure S4 Cross section of 3D synthetic structure with pore size threshold applied and without, Pt particles are more concentrated in certain areas when threshold is applied.

Wetting mechanism effects in partially wetted C only structure and SAXS profiles

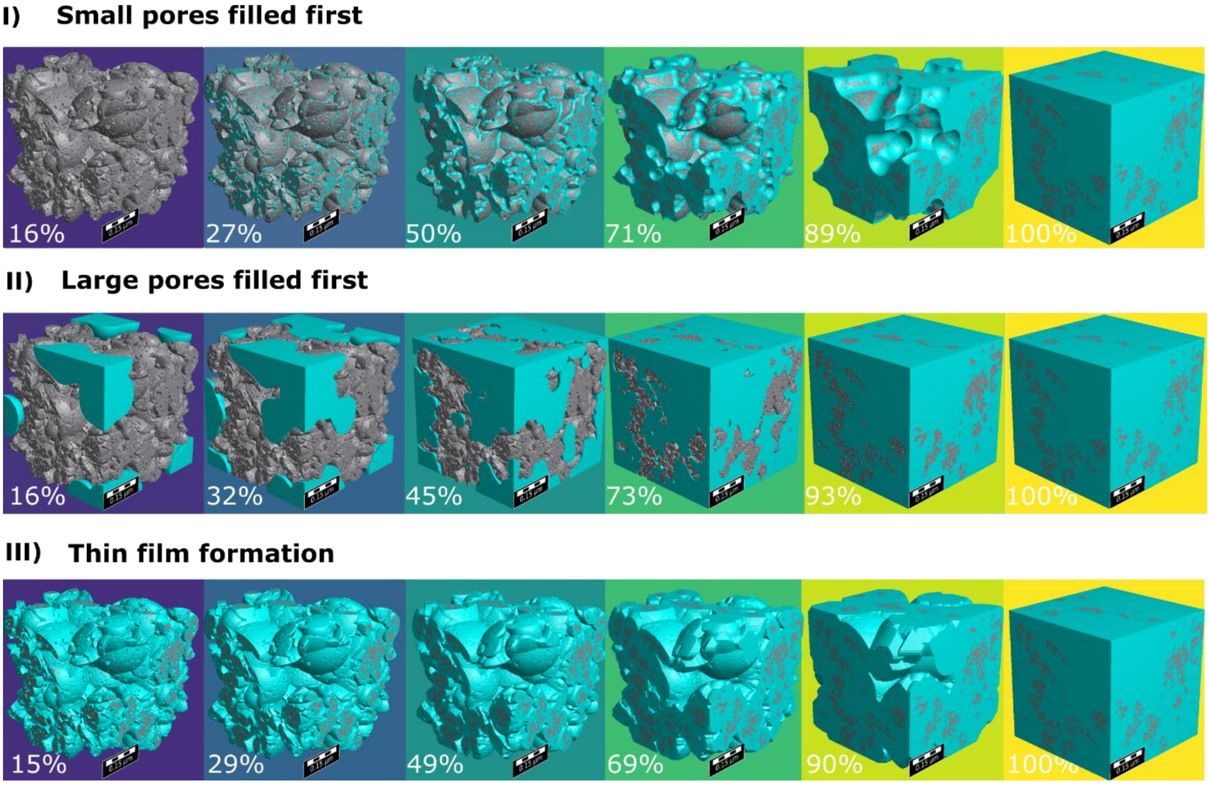

Figure S5 The different mechanisms of wetting in the pores of C only structure (intermediate towards Pt/C): I) smaller pores filling first, II) larger pores filling first, II) thin film formation.

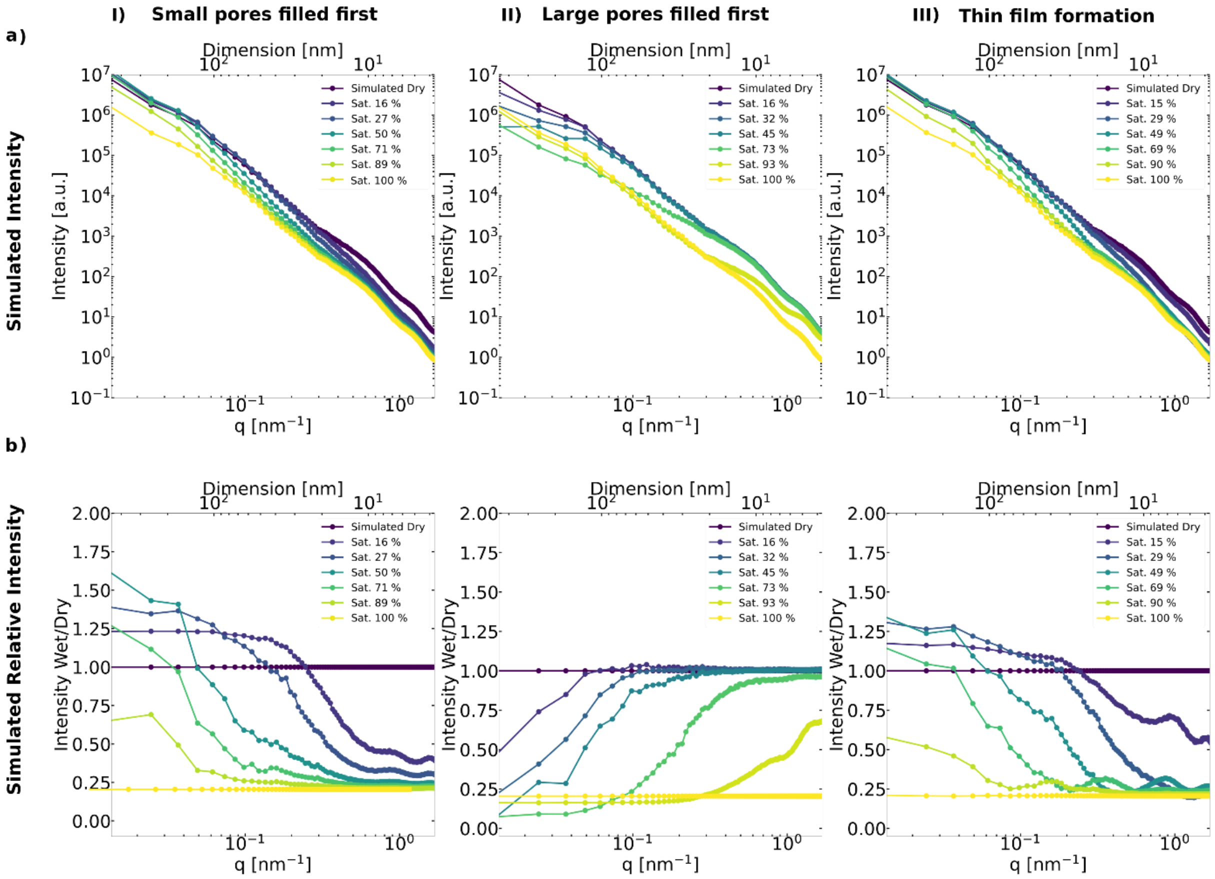

Figure S6 Simulated SAXS profiles of carbon only structure of different saturation levels in the three wetting mechanisms explored, and its corresponding relative intensities (wet/dry) to visualize better the intensity change (b-I-III). Color coded to the 3D structures in Figure S5.

Radiation damage test to dry sample with continuous *X*-ray exposure

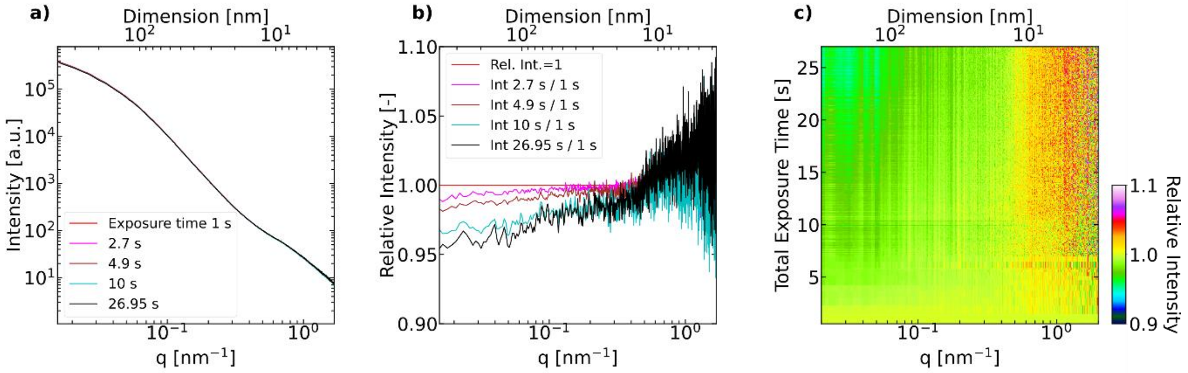

Figure S7 a) Intensity profiles at varying total exposure time, measurement time corrected to uncover morphology changes. b) Relative intensities at varying total exposure time (intensity

normalized by intensity at 1 s total exposure time). c) Relative intensities at finer total exposure time steps.

In order to assess the morphological stability of the Pt/C catalyst layer, a multitude of SAXS measurements were implemented on one spot of the dry electrode. The probed location accumulated a total *X*-ray exposure of 26.95 s, which is more than twice higher than the accumulated exposure of the individual measurement spots during the 10-minute *in situ* wetting experiment. While the log-scale representation of the intensity profiles are virtually overlapping (see Figure S7a), relative intensity changes (normalized by the intensity after 1 s exposure, see Figure S7b) reveal that there is an observable decrease in intensity at lower q (max -5%) and increase at higher q (up to +10%). The effect of *X*-ray exposure can be seen more detailed in Figure S7c, showing a general increase (red area) in high-q and intensity decrease (green area) at low-q with higher exposure time. The changes in *X*-ray scattering are caused by the extensive effects of the *X*-ray irradiation, including chain scission and cross-linking on the molecular level *(high q)* that lead to ionomer decomposition, thinning and detachment of the ionomer on the electrode level *(low q)* [2, 3]. The morphological changes due to *X*-ray irradiation are most likely also occurring during the *in situ* experiment and will mistakenly contribute to the saturation level change. The quantification of the morphological changes after an exposure time of 27 s by Invariant calculation results into a potential wrongful contribution to the saturation level changes of 6.9%.

Particle size effect during *in situ* potential hold and cycling

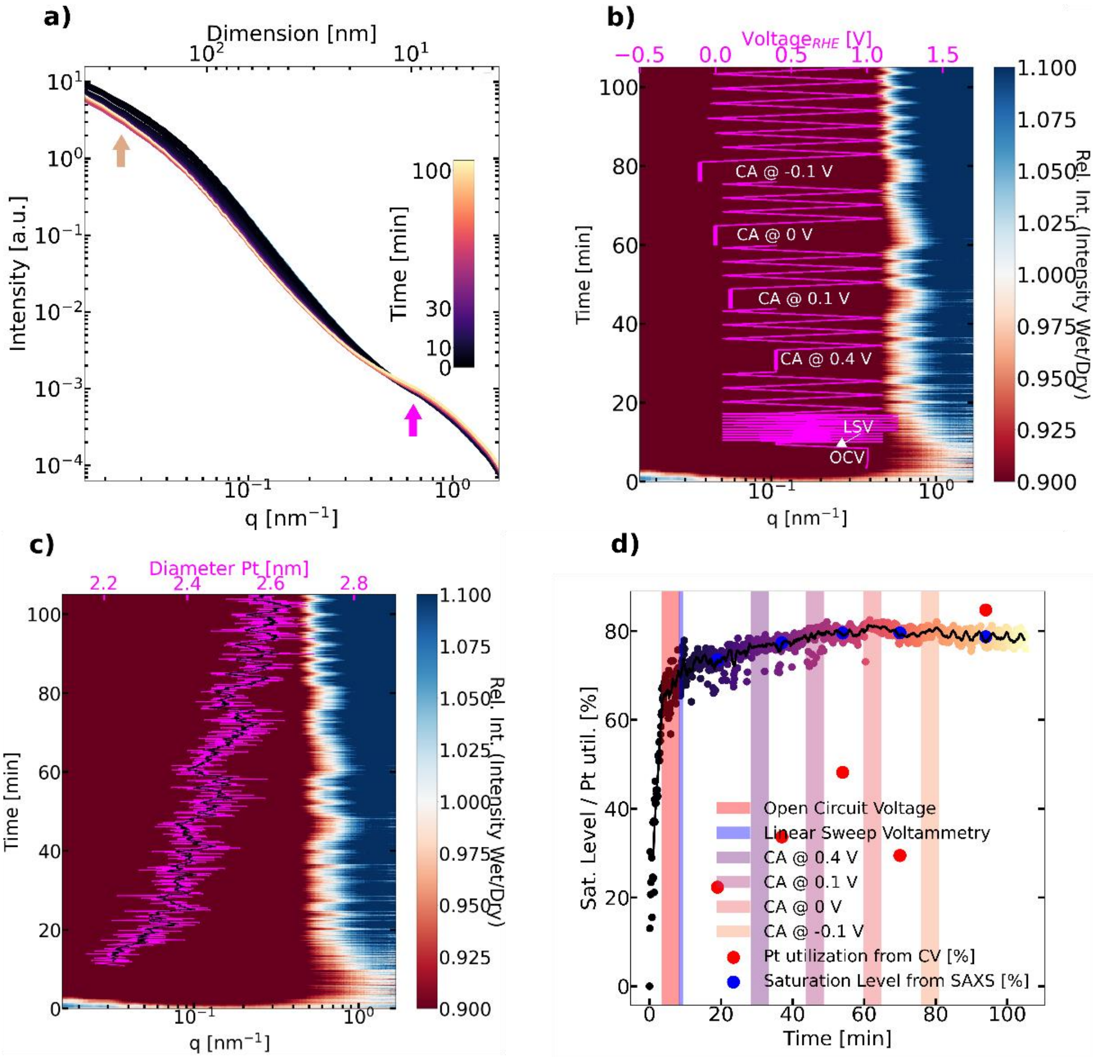

Figure S8 a) SAXS profiles taken during in situ wetting of Pt/C catalyst layer for the whole duration of the experiment, b) relative intensity (wet/dry) versus time and voltage versus time (top axis, pink), c) relative intensity (wet/dry) versus time and Pt diameter from analytical fit, d) saturation level calculated from Invariant calculation overtime versus Pt utilization from CV H_{upd}.

During the *in situ* experiment in the flow cell, the full SAXS intensity data was recorded for ~100 minutes. In the beginning, the open-circuit-voltage (OCV) was measured and was followed by the recording of a linear sweep down to 0.05 V_{RHE}. Cyclic voltammetry (CV) was

carried out in between chronoamperometric (CA) measurements at increasingly negative potentials to evolve hydrogen (specifically at -0.1 V_{RHE}) and therewith reduce the Pt and increase its utilization. From the SAXS profiles, an increase in intensity at higher-*q* can be observed with time (see Figure S8a).

Oscillations of the signal from high-*q* with respect to the potential are more clearly revealed by relative intensity plot in Figure S8b. These oscillations may stem from the formation and subsequent reduction of oxide depending on the applied potential during the CVs between 0.05 V_{RHE} and 1.1 V_{RHE} [4]. With increasing time and increasing number of cycles, the location of intensity increase (indicated by the pink arrow in Figure S8a) shifts to lower-*q*. This shift may originate from the increase of Pt size due to Oswald ripening [5] or the dissolution of smaller Pt nanoparticles and subsequent preferential redeposition on larger Pt nanoparticles. Continuous *X*-ray exposure may also speed up the Pt growth as seen in the radiation damage test, minor intensity increase at higher *q*.

Since the Pt contribution to the liquid water saturation level determination is found to be significant, before calculating the invariant to determine the saturation level of liquid water, a careful extraction of the constant contribution of Pt is needed. A fit to the Pt region ($0.6 < q < 1.5$ nm^{-1}) of the *in situ* SAXS profile to the analytical solution of sphere form factor with log-normal distribution was carried out using the JScatter Python package [6] by assuming that the pores near the Pt are fully filled or in other words assuming the contrast change is from particle size only. This may not be the case during the potential holds at 0 V and -0.1 V, as H_2-bubbles may have been produced near the Pt's surface, hence the particle size change may also hide small nanobubble development. The mean diameter of the Pt size increased from ~2.2 to 2.6 nm (see Figure S8c), mean diameter from the fit in pink and moving average in black). By subtracting the contribution of Pt using the analytical fit result, a relatively constant saturation level throughout the electrochemical procedure was observed (see Figure S8d).

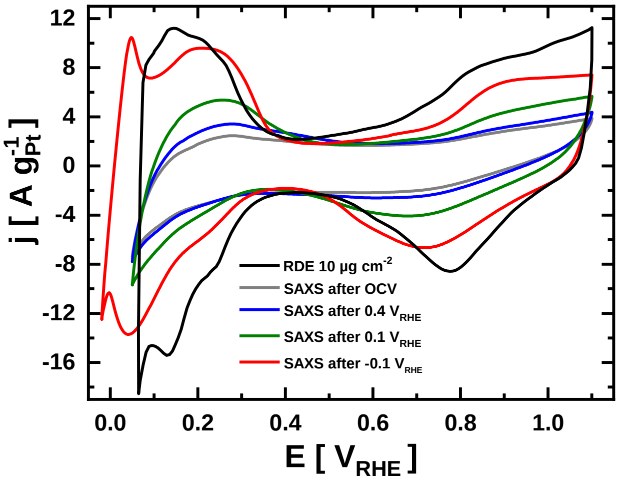

Figure S9 Cyclic voltammetry performed while measuring wetting of catalyst layer with SAXS.

Before the potential hold, the Pt utilization derived from CVs in Figure S9 indicated as red dots in Figure S8d was only 22.3%. After the potential hold at 0.4 V, the Pt utilization increased to 34%. Thereafter, the utilization further increased to 48.2% and 84.8% after holding potential at 0.1 V_{RHE} and -0.1 V_{RHE}. Meanwhile, the low utilization value of 29.4% found at 0 V_{RHE} is attributed to hydrogen bubble formation and due to the shielding of the surface under gas evolving conditions with limited mass transport as described in previous studies [5, 7, 8]. The formation of a bubble can be further proven by the increase in transmission intensity of the *X*-ray beam (Figure S10) and an intensity increase at low q (indicated with the brown arrow in Figure S8a) during the potential holds at 0 and -0.1 V_{RHE}. The transmission value does not fully recover during the CVs, indicating the electrolyte being replaced by gas leading to a lower absorbance. In contrast, the saturation levels from SAXS data (indicated by blue dots) are 73.6%, 77.3%, 79.6%, 79.6%, 78.7 % (after LSV, after 0.4 V, after 0.1 V, after 0 V, after -0.1 V, respectively) which remain relatively constant throughout, indicating that the wetting does not correlate with Pt utilization. The increase of Pt utilization may result from the cleaning of the Pt surface.

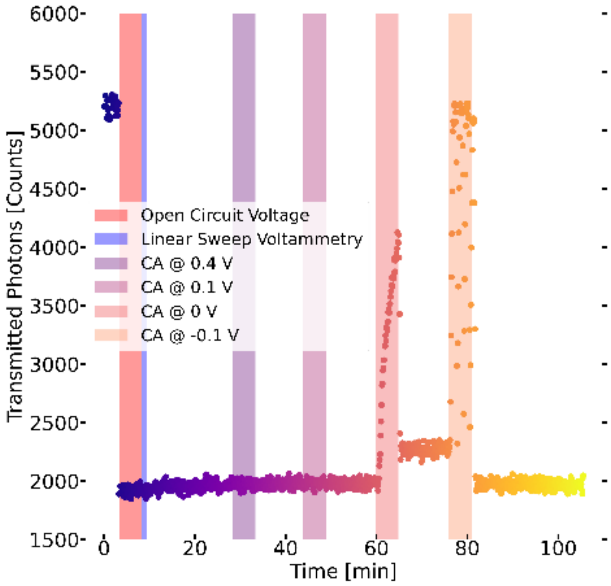

Figure S10 Transmitted counts versus time following the in situ wetting of the catalyst layer

Details on the IMG2SAS routine

IMG2SAS is a routine in FCSAXS package used to convert 3D isotropic image of scattering length density data to 1D SAXS intensity profile.

After applying a 3D FFT to the 3D image, the zero frequency of the resulting 3D FFT data is shifted to the center of the image. The 3D FFT arrays consist of real and imaginary parts. The 3D array of SAXS intensity is obtained by the squared magnitude of the 3D FFT array. The resulting of 3D intensity array was then subjected to spherical averaging of intensities with the same distance to the center creating a 1D intensity array (intensity versus distance to the center of the 3D array). The distance from cube's center can be related to reciprocal space,

$q_{min} = \frac{2\pi}{simulation\ box\ size * one\ voxel\ length\ in\ real\ space}$ and

$q_{max} = \frac{\pi}{one\ voxel\ in\ real\ space}$, with spacing between points of q equals to q_{min}.

The 1D simulated intensity profile then was convolved with a sinc function to reduce artifacts in the high-q due to the cubic voxel structure. The resulting intensity is in units of length^{-1}, with

the simulation box in units of length. For example, if the voxel size is user-defined as 1 nm, the intensity will have unit of nm^{-1}. It is to note that by doing spherical averaging, the resulting intensity profile is equivalent to having intensities averaged from all potential orientations of the sample relative to the *X*-ray beam, thus eliminating, or averaging out any anisotropy in the reciprocal-space map or in the 3D structure. This averaging is justified by observing that the SAXS patterns from the Pt/C catalyst layer are isotropic.

IMG2SAS verification

In order to validate IMG2SAS we applied it to functions for which an analytical solution is known, namely a spherical form factor, core-shell form factor, and a cubic form factor. These geometries were created with raster-geometry Python package. The resulting profiles from IMG2SAS are then compared to the analytical solution from JScatter Python package.

For the spherical form factor, the simulation with IMG2SAS is limited to the simulation box size used (denoted as a) and voxel size (denoted as v). Figure S11 shows the result for the form factor of spheres with different parameters of the simulation box size in nm, voxel size in nm, radius in nm, electron density contrast against air/vacuum in nm^{-2} (eta).

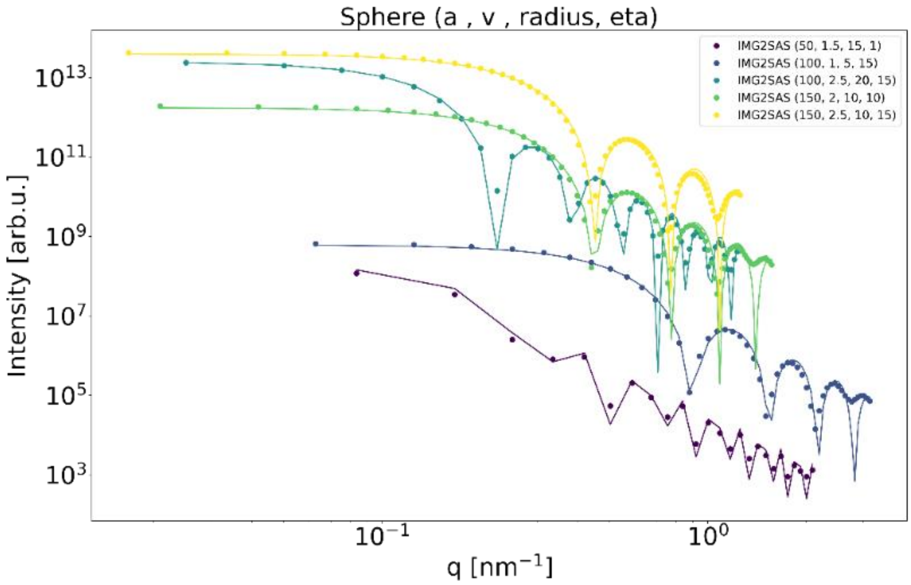

Figure S11 Simulated SAXS profiles obtained by IMG2SAS (dots) for spheres compared to the analytical solutions (lines); parameters in the brackets of the legend entries are IMG2SAS (box size in nm, voxel size in nm, radius in nm, electron contrast difference in nm^{-2}

Results for simulated SAXS profiles for a core and a shell are shown in Figure S12, for variations of the core radius, the shell thickness, as well as the scattering length densities of both.

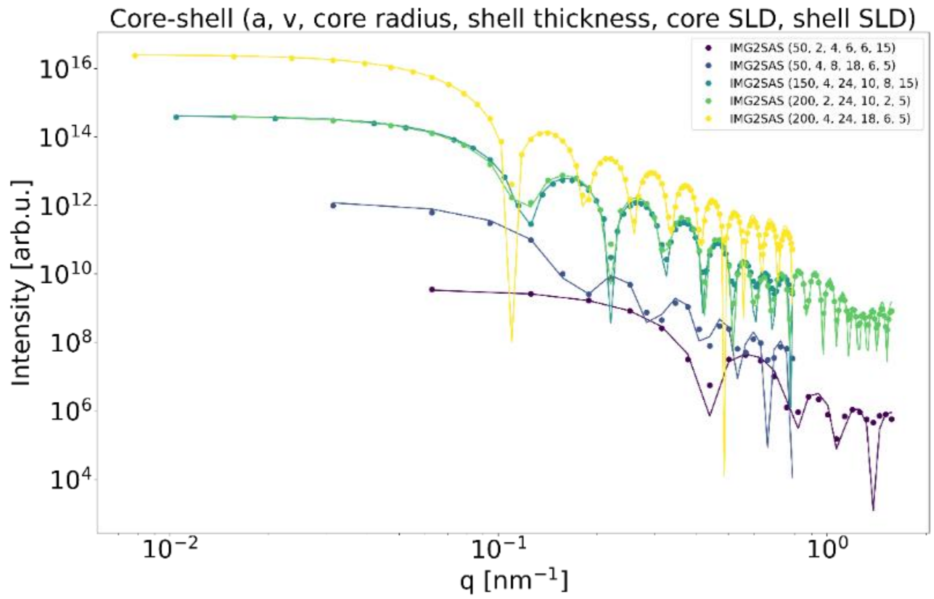

Figure S12 Simulated SAXS profiles (dots) obtained by IMG2SAS for core-shell spheres compared to analytical solutions (lines); parameters in the brackets of the legend entries are IMG2SAS(box size in nm, voxel size in nm, core shell radius in nm, shell thickness in nm, core SLD in nm^{-2}, shell SLD in nm^{-2}).

Results for simulated SAXS profiles cubes are show in Figure S13, where the simulation code receives different edge length of the cubes.

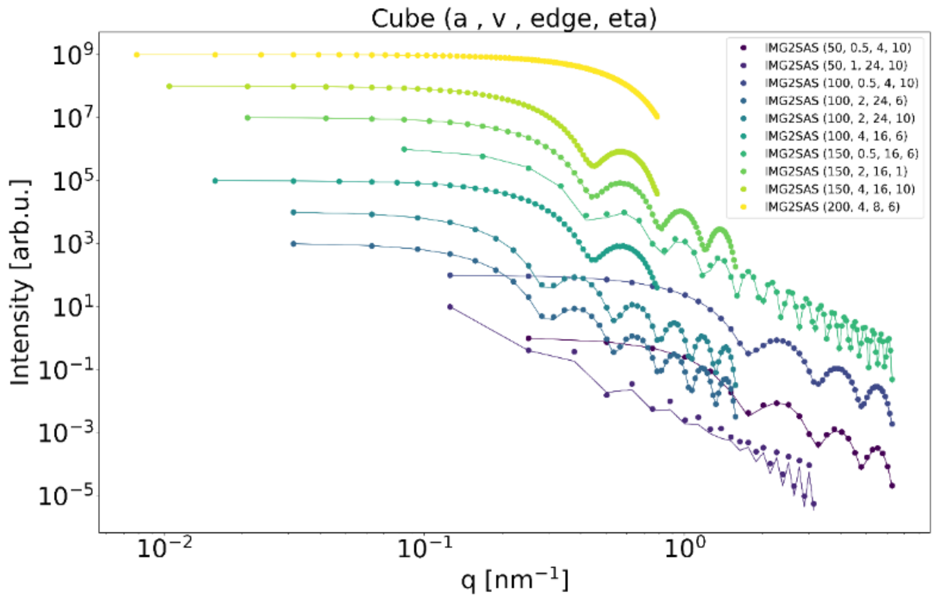

Figure S13 Simulated SAXS profiles (dots) obtained by IMG2SAS for cube compared to analytical solutions (lines); parameters in the brackets of the legend entries are IMG2SAS (box size in nm, voxel size in nm, edge length in nm, electron contrast difference in nm^{-2}).

For anisotropic objects such as cylinders, the resulting form factor from IMG2SAS is comparable to the analytical solution when the cylinder is averaged from all directions, as shown in Figure S14.

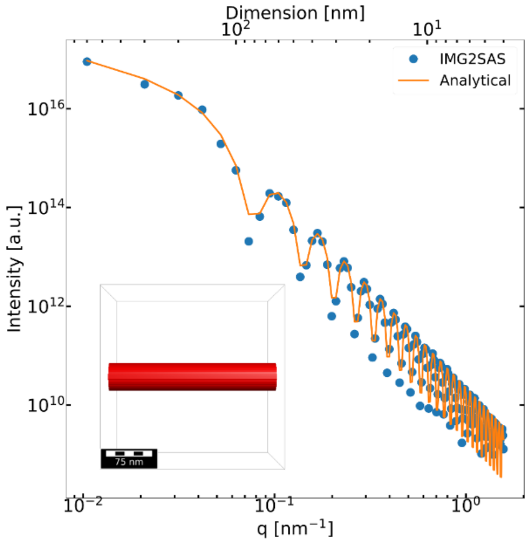

Figure S14 Simulated SAXS profile (dots) obtained by IMG2SAS for a cylinder compared to the analytical solution (line).

To demonstrate the applicability of IMG2SAS to more complex structures, an artificial porous media (see Figure S15a) was generated with the Porespy package [9] with different pore sizes with the Union Boolean Model (UBM) approach. The created structure was then characterized with the granulometry procedure of Geodict2021 to calculate pore size distribution as shown in Figure S15b. The SAXS analytical solution of the UBM is compared to the simulated numerical SAXS from the 3D voxel representation by IMG2SAS in Figure S15c, showing a good agreement for the accessible q-range of the IMG2SAS result.

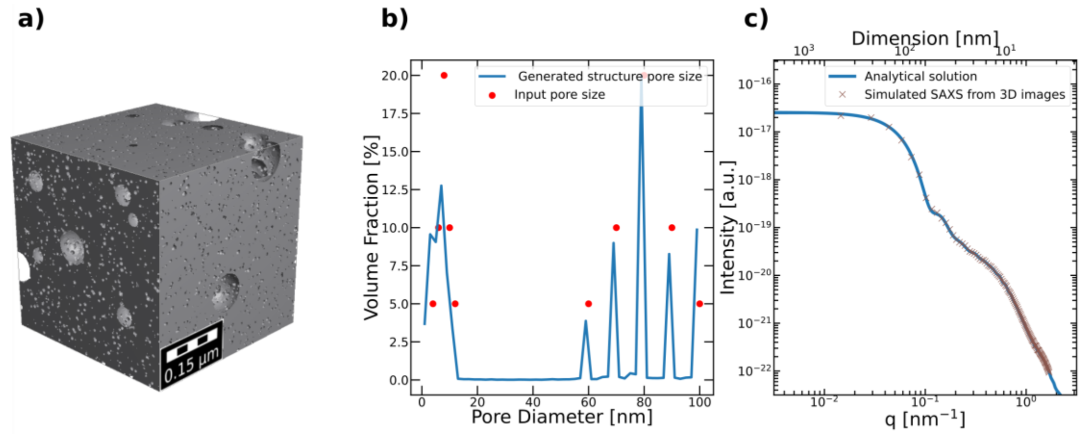

Figure S15 a) generated pore structure with overlapping spheres algorithm (UBM), b) the input parameters compared to the pore size distribution of the generated structure, c) comparison of SAXS analytical solution of overlapping/union Boolean model and its simulated SAXS from the 3D voxel representation by IMG2SAS.

Aperiodic structure effects in IMG2SAS

The 3D FFT algorithm that is used in IMG2SAS (and 3D FFT in general) assumes periodicity of the structure. However, in this manuscript the structures are not always periodic due the pore geometry and the water structure therein. In this section, two methods to deal with aperiodicity and edge effects are presented: 1) padding of the 3D structure plus mirroring along edge axes

and 2) window function to taper the edges of the 3D structure so that the edge values equal to 0.

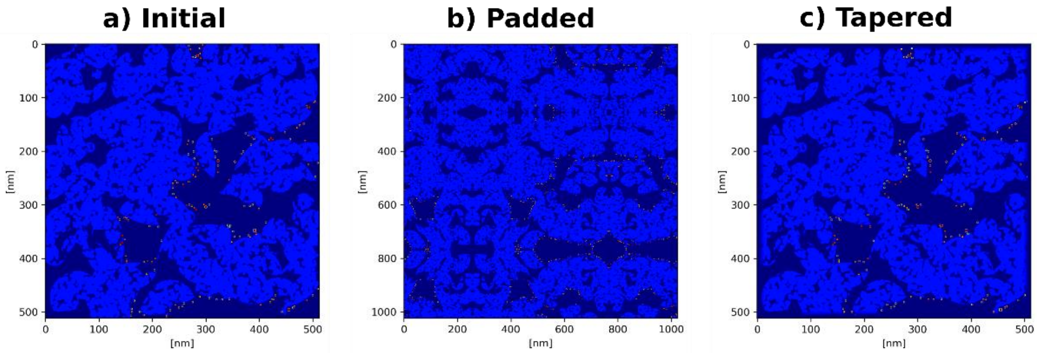

Figure S16 2D Cross section of a) an initial CL structure that undergoes modifications to eliminate boundary effects: b) padded and c) tapered.

Padding the structure results in an eight times larger structure compared to the initial structure (see Figure S16b compared to S16a), hence causing longer computation time. The padding method has an advantage over windowing as the porosity and hence the saturation level of the initial structure are conserved. The intensity profiles corresponding to the initial structures in Figure 6 with padding treatment reveals a similar trend to the structure without padding for the proposed wetting scenarios (see Figure S17) as well as similar saturation level by Invariant calculation in Figure S19a.

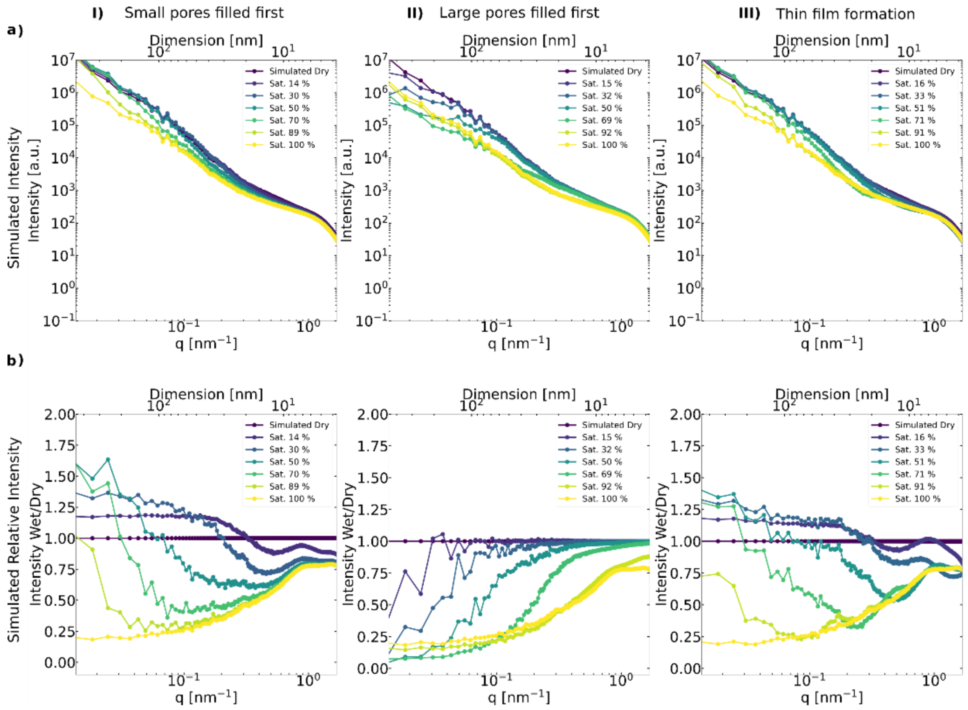

Figure S17 a) Simulated intensities and b) simulated relative intensities for 3 water filling mechanisms for the padded 3D structures. The corresponding initial structures are shown in Figure 6.

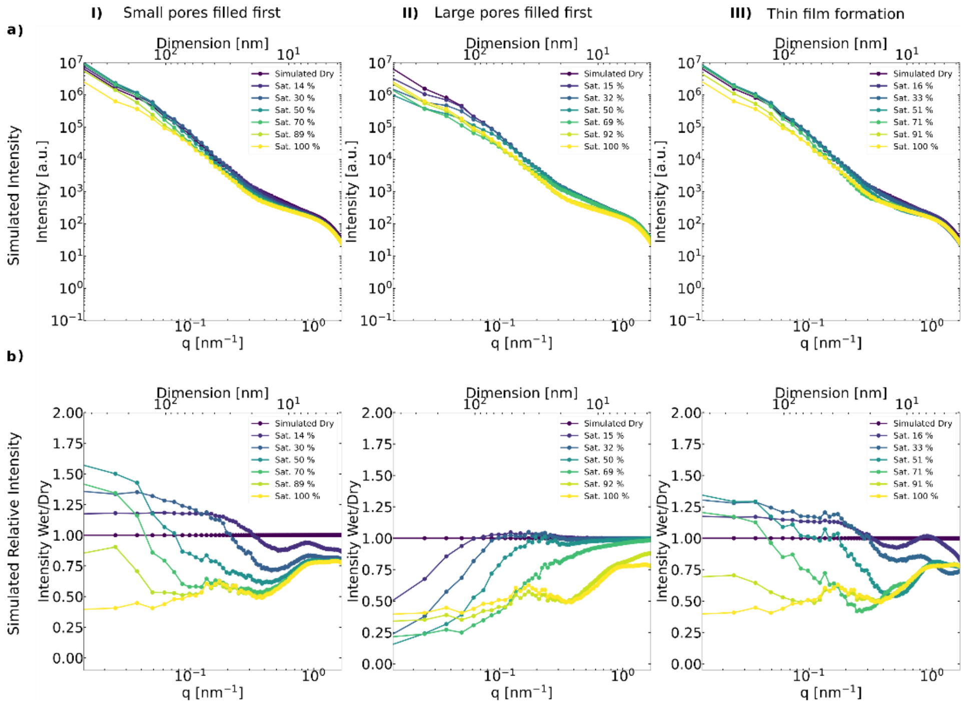

Figure S18 a) Simulated intensities and b) simulated relative intensities for 3 water filling mechanisms for tapered 3D structures. The corresponding initial structures are shown in Figure 6.

The alternative is to taper the edges of the 3D structure, modifying the initial electron density values. A tapered cosine window with 5% of the 3D structure in the boundary region is chosen as an example (see Figure S16c). The trend of intensity change during wetting is recovered by using the tapered structure (see Figure S18). However, since tapering modifies the electron density, the intensity profiles are different from the initial structures – particularly at low q, where the intensity profiles seem smoother. The discrepancy is also reflected in the obtained saturation levels (see Figure S19b) and mainly affects the large saturation levels. Hence, the usage of tapering is not recommended.

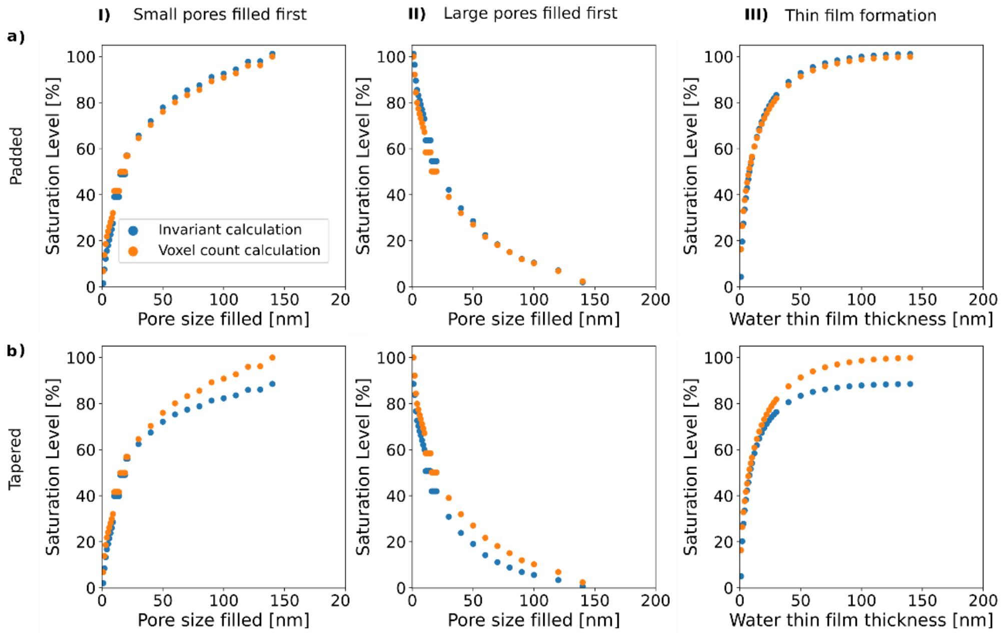

Figure S19 Saturation levels from voxel counting and Invariant for a) padded and b) tapered structures.

Extrapolation effects to the saturation levels obtained from the Invariant calculation

The Invariant in this manuscript is calculated by integration from q_{min} to q_{max} constrained by the experimental setup, whereas Invariant calculation ideally would be done by integrating from $q = 0$ to $q = \infty$. Extrapolation of the experimental to an extended q-range seems attractive to overcome this problem but needs to be done with great care. Extrapolation of the low q by fitting several points at low q where the intensity is plateauing by 1) Power law, 2) Guinier law, 3) constant value of the last point is carried out to determine potential saturation level calculation errors in respect to the saturation level calculated without extrapolation. The difference between saturation levels during the *in situ* wetting experiment for first 10 minutes obtained by the three extrapolation methods and without are within ≈ 1-2% for each value. The negligible influence of the extrapolation for smaller q on the Invariant based saturation validates that the experimental q-range is sufficient to determine the saturation of in the pores of the CLs in PEFCs.

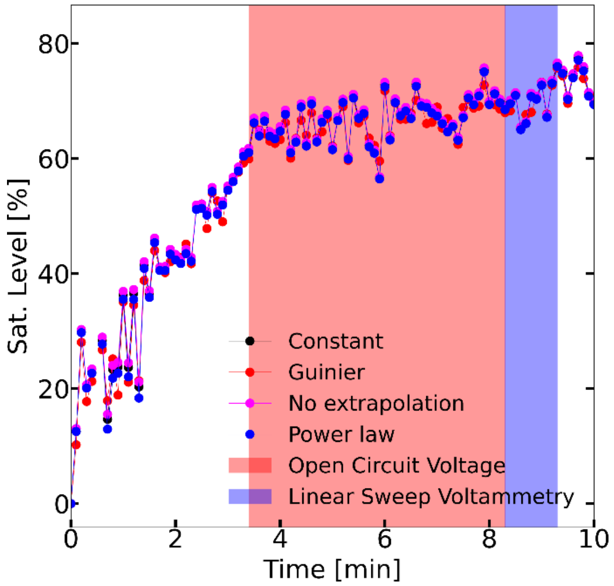

Figure S20 Saturation levels of Figure 9c compared with the ones obtained from three methods for low-*q* extrapolation 1) Constant, 2) Guinier, 3) Power law.

Additional statistical measures of reconstructed CL from SAXS

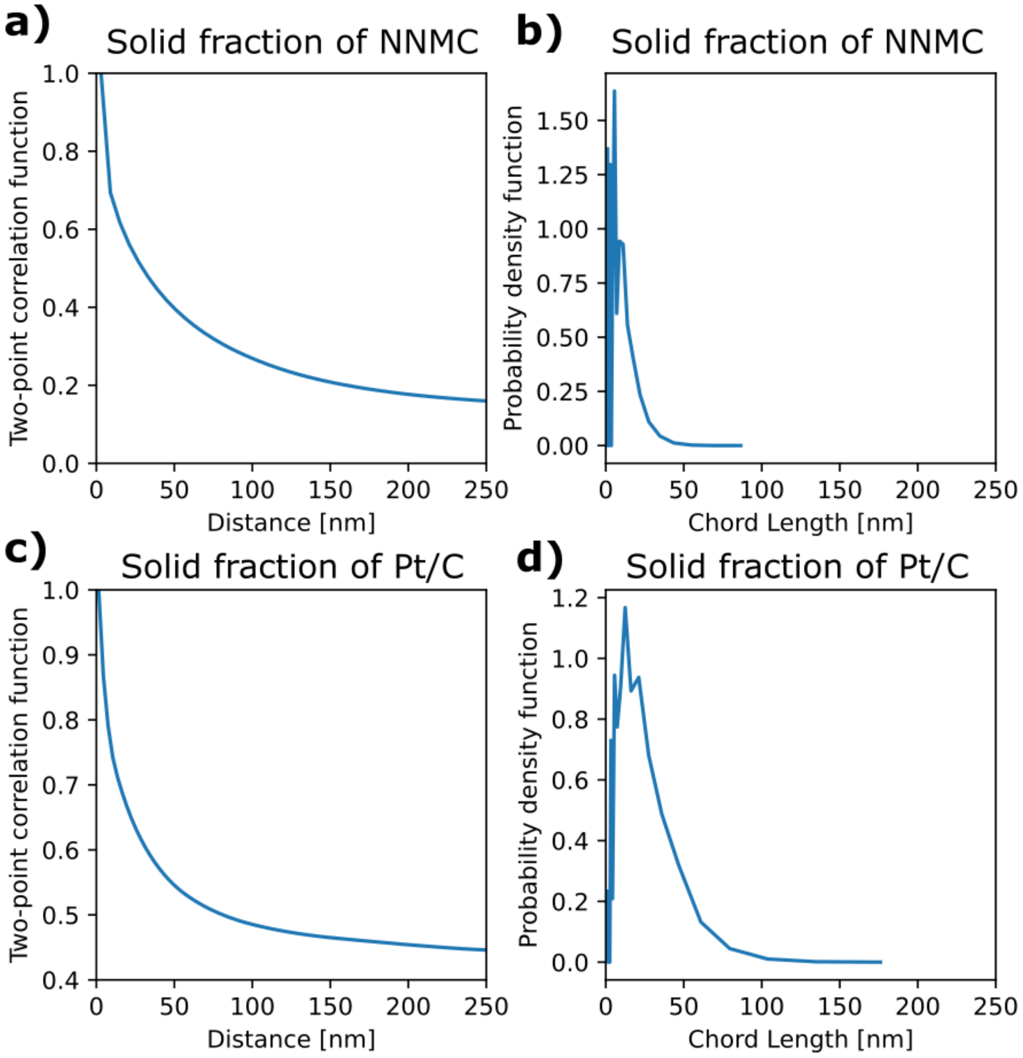

Figure S21 a) Two-point correlation function for the solid fraction of NNMC and Pt/C c). b) Chord length distribution for the solid fraction of NNMC and Pt/C d).

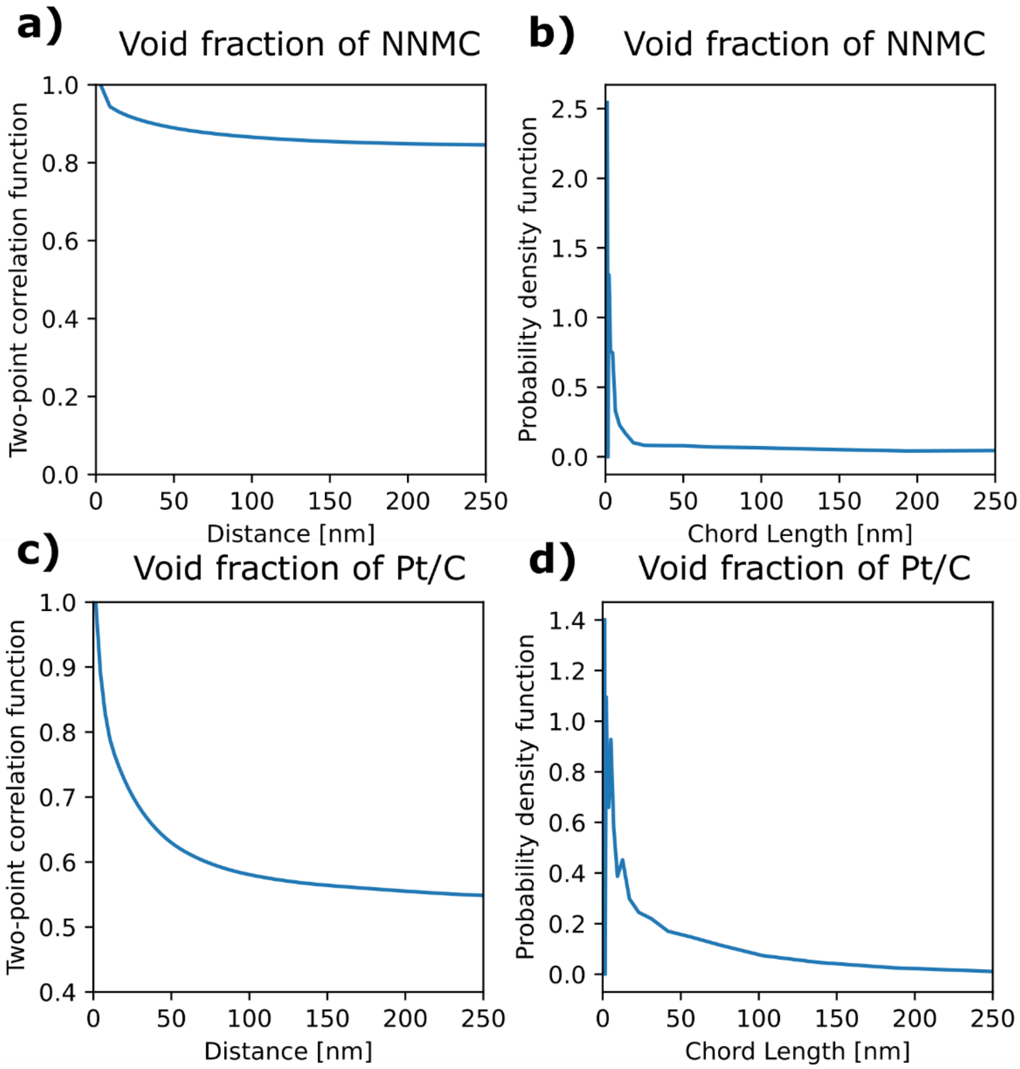

Figure S22 a) Two-point correlation function for the void fraction of NNMC and Pt/C c). b) Chord length distribution for the void fraction of NNMC and Pt/C d).

REFERENCES

(1) Binninger, T.; Fabbri, E.; Patru, A.; Garganourakis, M.; Han, J.; Abbott, D. F.; Sereda, O.; Kotz, R.; Menzel, A.; Nachtegaal, M.; et al. Electrochemical Flow-Cell Setup for in Situ X-Ray Investigations I. Cell for SAXS and XAS at Synchrotron Facilities. *J Electrochem Soc* **2016**, *163* (10), H906-H912. DOI: 10.1149/2.0201610jes.

(2) Roth, J.; Eller, J.; Buchi, F. N. Effects of Synchrotron Radiation on Fuel Cell Materials. *J Electrochem Soc* **2012**, *159* (8), F449-F455. DOI: 10.1149/2.042208jes.

(3) Kulkarni, D.; Normile, S. J.; Connolly, L. G.; Zenyuk, I. V. Development of Low Temperature Fuel Cell Holders for Operando X-Ray Micro and Nano Computed Tomography to Visualize Water Distribution. *J Phys-Energy* **2020**, *2* (4). DOI: ARTN 044005 10.1088/2515-7655/abb783.

(4) Haubold, H. G.; Wang, X. H.; Goerigk, G.; Schilling, W. In Situ Anomalous Small-Angle X-Ray Scattering Investigation of Carbon-Supported Electrocatalysts. *J Appl Crystallogr* **1997**, *30* (2), 653-658.

(5) Povia, M.; Herranz, J.; Binninger, T.; Nachtegaal, M.; Diaz, A.; Kohlbrecher, J.; Abbott, D. F.; Kim, B. J.; Schmidt, T. J. Combining SAXS and XAS to Study the Operando Degradation of Carbon-Supported Pt-Nanoparticle Fuel Cell Catalysts. *ACS Catal* **2018**, *8* (8), 7000-7015.

(6) Biehl, R. Jscatter, a Program for Evaluation and Analysis of Experimental Data. *Plos One* **2019**, *14* (6). DOI: ARTN e0218789 10.1371/journal.pone.0218789.

(7) Diklic, N.; Clark, A. H.; Herranz, J.; Diercks, J. S.; Aegerter, D.; Nachtegaal, M.; Beard, A.; Schmidt, T. J. Potential Pitfalls in the Operando XAS Study of Oxygen Evolution Electrocatalysts. *ACS Energy Lett* **2022**, *7* (5), 1735-1740. DOI: 10.1021/ACSenergylett.2c00727.

(8) Diercks, J. S.; Pribyl-Kranewitter, B.; Herranz, J.; Chauhan, P.; Faisnel, A.; Schmidt, T. J. An Online Gas Chromatography Cell Setup for Accurate Co2-Electroreduction Product Quantification. *J Electrochem Soc* **2021**, *168* (6). DOI: ARTN 064504 10.1149/1945-7111/ac0363.

(9) Gostick J.; Khan Z. A. ; Tranter T.G.; Kok M. D. R.; Agnaou M.; Sadeghi M. A.; R., J. Porespy: A Python Toolkit for Quantitative Analysis of Porous Media Images. *Journal of Open Source Software* **2019**, *4* (37).
